# Solid-phase-supported synthesis of morpholinoglycine oligonucleotide mimics

**DOI:** 10.3762/bjoc.10.115

**Published:** 2014-05-20

**Authors:** Tatyana V Abramova, Sergey S Belov, Yulia V Tarasenko, Vladimir N Silnikov

**Affiliations:** 1 Institute of Chemical Biology and Fundamental Medicine, SB RAS, Lavrent’ev Ave, 8, Novosibirsk 630090, Russia; 2 Novosibirsk State University, Pirogova St. 2, Novosibirsk 630090, Russia

**Keywords:** labile linker, morpholino oligomers, oligonucleotide mimics, solid-phase-supported peptide synthesis (SPPS)

## Abstract

An efficient solid-phase-supported peptide synthesis (SPPS) of morpholinoglycine oligonucleotide (MorGly) mimics has been developed. The proposed strategy includes a novel specially designed labile linker group containing the oxalyl residue and the 2-aminomethylmorpholino nucleoside analogues as first subunits.

## Introduction

The phosphorodiamidate morpholino oligomers (PMO) and peptide conjugated PMO (PPMO) are currently promising candidates for antisense therapy of a number of infectious and hereditary diseases [1–4] despite of some difficulties and limitations. They also proved to be valuable tools to study fundamental problems of gene expression in the course of embryogenesis [5–6]. A few examples of morpholino oligomers containing other types of internucleoside bonds were described. An attractive feature of these new morpholino oligonucleotide analogues is the absence of additional chiral centers. Unlike commercially available PMO and PPMO, which are mixtures of diastereomers, oligomers constructed with the use of phosphoromonoamidate [7], oxalyl diamide [8], amidine [9] and methylene caxboxamide [10] groups represent the individual compounds. It means that their physicochemical properties, such as thermostability of complementary complexes, do not depend on the diastereomeric composition of the mixtures, which can vary in different preparations. The difference in the spatial structure, in the extent of base stacking, and in the stability of complementary duplexes for individual diastereomers has been demonstrated for phosphotriester [11] and methylphosphonate [12] oligonucleotide analogues and PMO [13].

The degree of protonation at physiological pH and the presence of positively charged centers in oligonucleotide analogues results in increased water solubility and higher thermal stability of complementary duplexes of such oligonucleotide mimics with nucleic acids [14–15]. Some conformationally restricted protonated PNA or pyrrolidine oligonucleotide mimics exhibit selective binding with RNA but not with DNA [16–17]. It has been shown that conjugation of PMO with cationic peptides or other cationic residues facilitates penetration of PMO into cells and efficiently prevents the growth of *E. coli* in vitro and in vivo [18].

**Figure 1:**
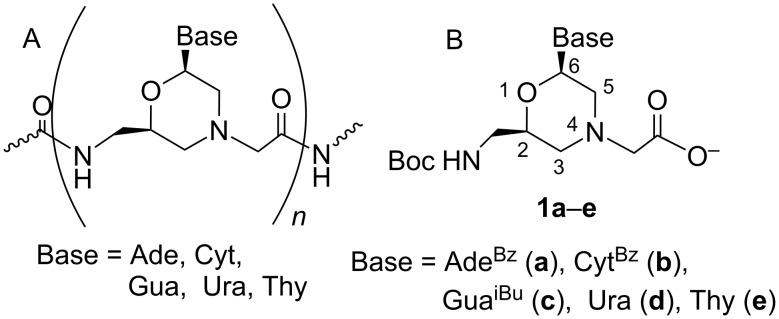
Morpholinoglycine oligomers (A) and protected monomers **1a**–**e** (B) for their synthesis.

Taking the above considerations into account, methylenecarboxamide (glycine) oligomers (MorGly) (Figure 1A), being protonated at physiological pH, seem to be promising candidates for the development of novel antisense oligonucleotide mimics.

**Figure 2:**
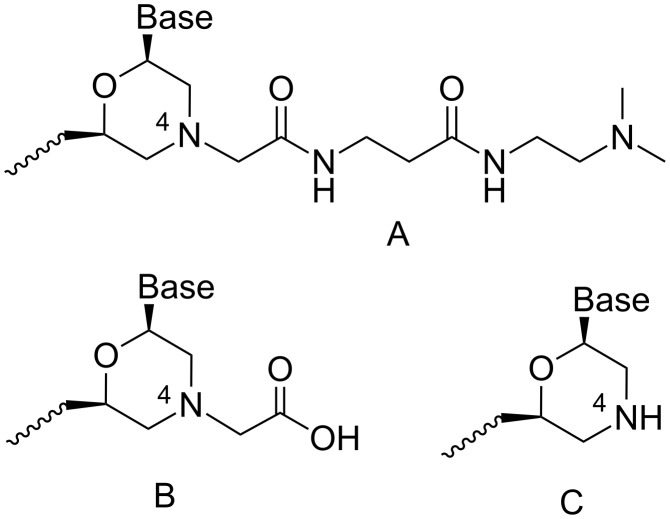
Dangling residues of MorGly oligomers synthesized by solid phase supported (A) and liquid phase synthesis (B); the blunt end of MorGly oligomer (C).

A convenient synthetic procedure was previously described for protected monomers **1a**–**d** (Figure 1B) necessary for the synthesis of the MorGly oligonucleotide mimics [19]. When studying the tandem complementary complexes of MorGly homohexa- and pentamers, we found out that the adenine-containing MorGly oligomers formed more stable complexes with poly(U) than native oligodeoxyriboadenylates of the same length. Moreover, it was revealed that the MorGly oligomers preferably bind with RNA than with DNA [20]. At the same time, the stability of complementary complexes of modified oligomers was shown to depend on the residues dangling the 4-end of the MorGly oligomers. In this study, the type of the residue dangling the 4-end of the oligomer chain (Figure 2) depended on the utilized synthetic method. We found that the MorGly oligomers containing the residues of type A formed less stable complementary complexes than oligomers containing residues of type B.

Another interesting fact demonstrated in the article [20] was the dependence of the thermal stability of complementary complexes formed by the MorGly oligomers on the heterocyclic base composition (uracil or adenine) of the modified chain. It was shown while studying their tandem complexes that the impact of cooperative interactions at oligomer junctions on the thermal stability was higher for modified oligomers than for native oligodeoxyriboadenylates. This result may indicate that the contribution of nucleobase stacking to the thermal stability of complementary complexes is more important for the oligonucleotide mimics than for native oligonucleotides. The substitution of the uracil nucleobases in the MorGly oligomers by thymines with better stacking properties may solve this problem. The synthesis of the thymine containing morpholino monomer **1e** was necessary to prove this hypothesis.

The promising properties of the MorGly oligomers as potential antisense agents motivated us to develop the SPPS method for the synthesis of these oligonucleotide mimics. We focused our efforts on the development of a new linker group to produce oligomers without dangling residues (Figure 2C), the synthesis of the corresponding Thy-containing monomers and the improvement of the SPPS protocol for the MorGly oligomers.

## Results and Discussion

For the synthesis of the thymine-containing monomer **1e** we applied the procedure published for obtaining **1d** [19] using 5-methyluridine as parent compound. Synthetic schemes, procedures and physicochemical data for intermediates in the synthesis of monomer **1e** are given in Supporting Information File 1.

**Figure 3:**
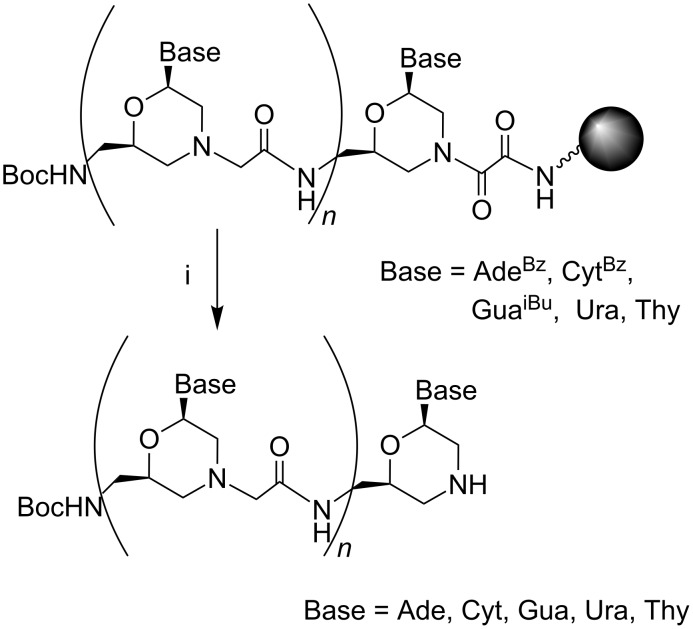
Cleavage of MorGly oligonucleotide mimics from solid support and deprotection of nucleobases by aqueous ammonia treatment: i) NH_3_/H_2_O.

We have previously shown that the morpholino oxalyldiamide oligomers were cleaved by aqueous ammonia treatment, which resulted in the formation of shortened oligomers with the blunt end of type C (Figure 2) [8]. So, the oxalyl residue can be used as a labile linker between the solid support and the growing oligomer chain (Figure 3) during SPPS in combination with the *tert*-butyloxycarbonyl (Boc)- and acyl-protected morpholino nucleosides.

After completion of the synthesis, the cleavage of the oligomer from the solid support and the deprotection of nucleobases can be performed simultaneously by treatment with aqueous ammonia as in the solid phase synthesis of native oligonucleotides (ODN) [21]. The oxalyl-mediated attachment of the growing chain to the solid support is usual in the synthesis of base sensitive ODN derivatives [22].

**Scheme 1:**
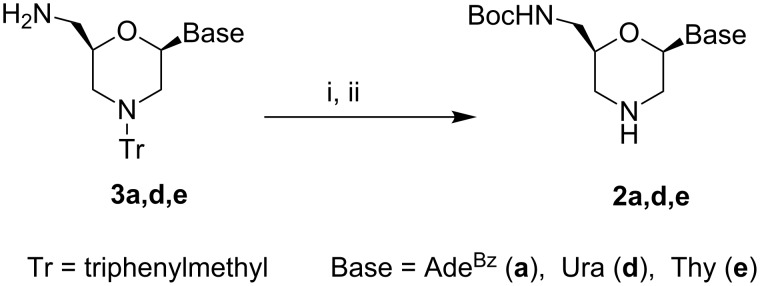
Synthesis of Boc-protected 2-aminomethylmorpholino nucleosides: i) di-*tert*-butyl dicarbonate ((Boc)_2_O), triethylamine (TEA), pyridine (Py); ii) AcOH/H_2_O.

Figure 3 indicates the necessity of using monomers **2** (Scheme 1) as first subunits bound to the support through the oxalyl linker. We synthesized Boc-protected aminomethylmorpholino nucleosides **2a,d,e** as shown in Scheme 1 starting from aminomethylmorpholino nucleosides **3a,d,e**. The synthesis of adenine and uracil containing compounds **3a,d** was published earlier [8]. Thymine containing morpholino nucleoside **3e** was obtained similarly to **3d** starting from the 5-methyluridine. See Supporting Information File 1 for the synthetic schemes, procedures and physicochemical data for intermediates in the synthesis of monomer **3e**.

**Scheme 2:**
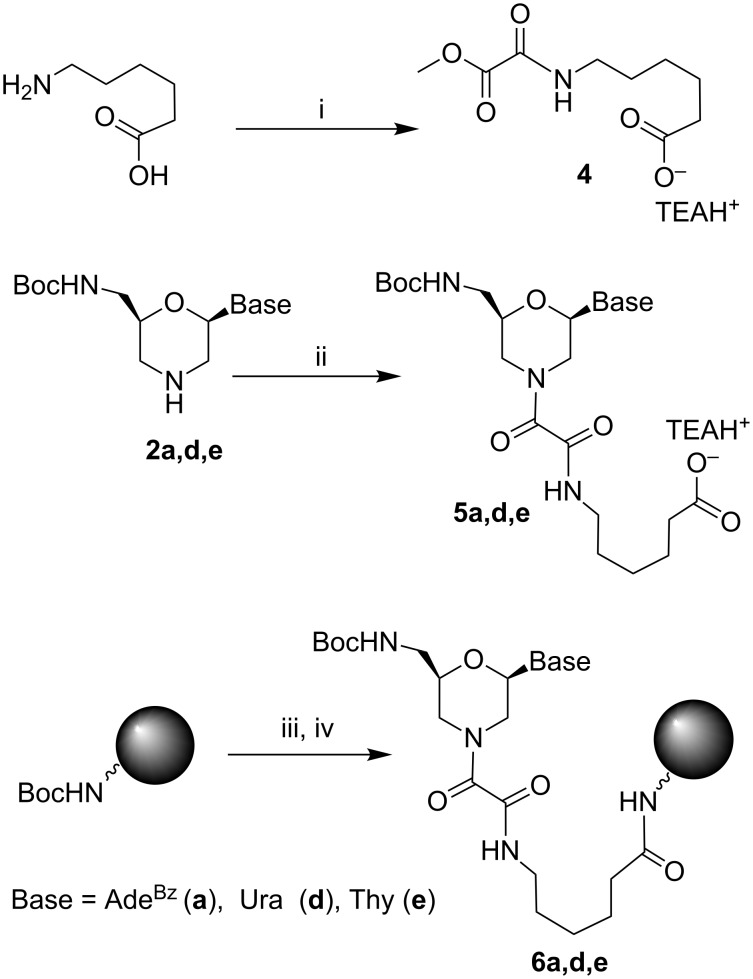
Loading of the Boc-Gly-PAM resin with the morpholino nucleosides **5a,d,e**: i) Dimethyl oxalate, TEA, MeOH; ii) **4**, TEA, Py; iii) trifluoroacetic acid (TFA)/CH_2_Cl_2_/PhSH; iv) coupling with **5a,d,e**. See Table 1 for detailed protocol.

Our next goal was to attach morpholino monomers **2a,d,e** to the Boc-Gly-PAM resin through the oxalyl linker. We tried different ways to do this including to change the order in assembling the elements of the whole construction, the direct attachment of the monomer unit to the support without additional 6-aminohexanoic acid and the application of more active bis(2-cyanoethyl)oxalate instead of dimethyl oxalate. However, only the route shown in Scheme 2 was successful and gave the loading of the polymer with the monomers **5a,d,e** close to that of the parent resin. It is interesting that the attachment of the oxalyl-containing residue to the morpholine nitrogen in monomers **5a,d,e** resulted in the appearance of duplicate signals of the nucleobases and morpholine protons in the NMR spectra (see Experimental) similar to morpholinooxalyl nucleosides [8].

After completion of the loading, the unreacted amino groups were capped by diethyl pyrocarbonate (DEPC) as described earlier [23]. The extent of the loading and the rate of the cleavage of loaded monomers were determined after the aqueous ammonia/iPrOH treatment of aliquots of the support. The capacity of loaded supports **6a,d,e** was achieved as much as 0.61–0.65 mmol/g after 4 h of the coupling reaction between monomers **5a,d,e** and the Boc-Gly-PAM polymer resin. It was found that the cleavage of the monomers from the solid support in the course of the ammonia treatment was completed within two days at room temperature (see Supporting Information File 1). The structure of the monomers obtained after the ammonia treatment (Figure 3, *n* = 0, Base = Ade, Ura, Thy) was confirmed by thin-layer chromatography (TLC), reversed phase chromatography (RPC), and mass spectrometry (see Supporting Information File 1).

The cleavable linker containing the S–S bond has been previously used in the synthesis of PMO in the 2→4 direction [23]. This corresponds to the 5’→3’ direction of native oligonucleotides. In our case, the MorGly oligomers were synthesized in the opposite direction (4→2) [20] similar to the standard direction (3’→5’) of native ODN and peptide nucleic acid (PNA) synthesis. Probably, the same S–S-containing linker could be used in the synthesis of MorGly oligomers, but it should be examined. In our work we propose another approach, which has fewer steps and does not require the additional thiol treatment to cleave the oligomer from the support.

In our previous work [20], the reaction yields of the monomer attachment to the growing chain have dropped to 50% after the two first couplings although the addition of the first and the second monomer units provided a good yield (95%). The change of the solvents in SPPS of MorGly oligomers (see Table 1) similarly to the synthesis protocol for PMO [23] allowed us to achieve high yields in the coupling reaction. The synthesis of MorGly pentamers was performed manually in the mini spin receiver columns (0.7 mL) according to the improved protocol of the SPPS of MorGly oligomers (Table 1). The receiver column was shaken to mix the support and reactants. Swelling and capping steps were omitted beginning the second cycle. After each cycle, a small sample of the support was subjected to aqueous ammonia treatment to analyse resulting oligomers. The homogeneity and the structure of the products formed after each cycle were confirmed by TLC, RPC and mass spectrometry before and after removal of the Boc protective group (see Supporting Information File 1 for details).

**Table 1:** SPPS protocol for MorGLy oligomers using Boc-Gly-PAM resin (10–30 mg).

Entry	Step	Volume	Reagents and solvents	Time

1	swelling	0.7 mL	1,3-dimethyl-2-imidazolidinone (DMI)	1 h
2	washing	2 × 0.5 mL	dichloromethane (DCM)	2 × 3 min
3	deprotection	2 × 0.5 mL	0.4 M PhSH in DCM/TFA, 1:4	2 × 10 min
4	washing	5 × 0.5 mL	DCM, DMI, DCM, DMI, DCM	5 × 3 min
5	neutralization	2 × 0.5 mL	1-methyl-2-pyrrolidinone (NMP)/*N,N*-diisopropylethylamine (DIPEA), 9:1	2 × 1 min
6	activation	0.04–0.12 mL	0.2 M **5a,d,e** at the first cycle, **1a,d,e** at subsequent cycles; 0.18 M *O*-(benzotriazol-1-yl)-*N,N,N′,N*′-tetramethyluronium tetrafluoroborate (TBTU); 0.18 M DIPEA; in DMI	5–8 min
7	coupling	0.04 mL of the solution of activated monomer per 10 mg of the resin	solution of activated monomer obtained at step 6	4 h
8	washing	4 × 0.5 mL	DMI, DCM, DMI, DCM	4 × 3 min
9	capping	2 × 0.3 mL	DEPC/DCM, 1:9	2 × 5 min
10	washing	2 × 0.5 mL	DCM	2 × 3 min

It is interesting to note that the mobility of the homooligomers in TLC decreased with increasing of their length (Table S1, Supporting Information File 1). This fact helped us to estimate the comparative length and purity of homooligomers. The yield in each cycle was 96–98%.

**Figure 4:**
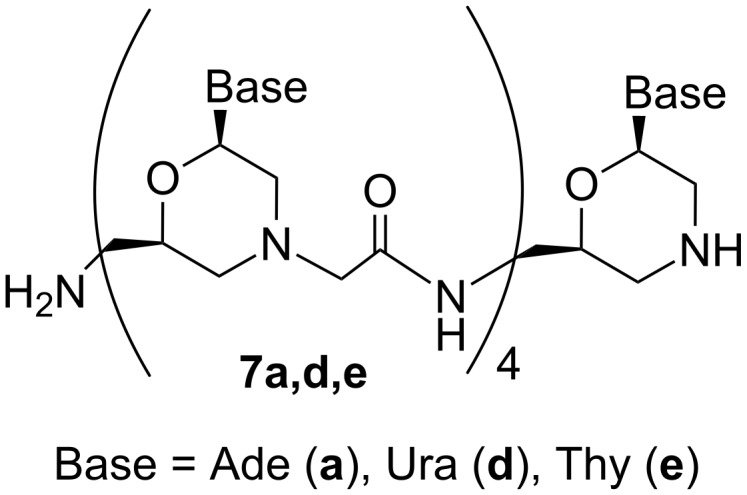
MorGly homopentamers containing adenine, uracil, and thymine nucleobases.

After completion of the pentamer synthesis and cleavage of the oligomer from the support, the Boc protective group was removed by trifluoroacetic acid (TFA). Target homopentamers **7a,d,e** (Figure 4) were purified by cation exchange chromatography and RPC.

## Conclusion

We developed a novel type of the labile linker for the synthesis of MorGly oligomers and improved the SPPS protocol for MorGly oligomers providing high yields at each cycle. Pentamers **7a,d,e** obtained by the proposed strategy will be used in the further study of the structure and the thermal stability of complementary complexes of the MorGly oligomers with native DNA and RNA to elucidate the role of the hydrogen bonds and base stacking in their formation and stability. The use of highly sophisticated constructs containing short modified oligonucleotides is nowadays a promising strategy in the antisense therapy [24]. Recently, short cationic morpholinoguanidinium oligomers were shown to penetrate living cells and to facilitate the inhibition of *Gli1* [25].

The unique properties of MorGly oligomers, such as the protonated backbone at physiological pH, stability to nucleases, and more preferable complex formation with RNA than with DNA, make them an attractive alternative in comparison to native oligonucleotides and other derivatives or analogues.

## Experimental

### General

We used 5-methyluridine, *O*-(benzotriazol-1-yl)-*N,N,N’,N’*-tetramethyluronium tetrafluoroborate, 1-methyl-2-pyrrolidinone, 1,3-dimethyl-2-imidazolidinone (Sigma-Aldrich, USA); Boc-Gly-PAM resin (substitution 0.76 mmol/g, NovaBiochem, Germany); sodium azide, glycine (Serva, Germany); sodium periodate, bromotrichloromethane, di-*tert*-butyl dicarbonate (Acros Organics, USA). Receiver columns (REF 740522, Macherey–Nagel, Germany) were used for SPPS of the MorGly oligomers in manual mode. All other reagents and solvents were from Sigma–Aldrich (USA) and Reachem (Russia). NMR spectra were recorded on a Bruker AV400 spectrometer (Bruker, Germany) in appropriate deuterated solvents at 30 °C. Chemical shifts (δ) are reported in ppm relative to TMS signals. Coupling constants *J* are reported in Hertz. MALDI–TOF mass spectra were registered on an Autoflex III mass spectrometer (Bruker Daltonics, Germany) using 2,5-dihydroxybenzoic acid as a matrix (MALDI–TOF) in positive or negative mode in The Center of Cooperative Use (“Proteomics”, Russian Academy of Sciences). IR spectra were recorded on a Vector 22 spectrometer (Bruker Optics, Germany) in KBr.

Quantitative analytical RPC were performed on a Milichrom A02 chromatograph system (Econova, Russia) on a ProntoSIL 125 C_18_ column (2 × 75 mm) in a gradient of buffer B (0.1 M TEA–AcOH, pH 7.0, 80% acetonitrile) in buffer A (0.1 M TEA–AcOH, pH 7.0, water) (0–100 % over 15 min) with an elution rate of 0.2 mL/min and UV detection at 250, 260, 280, and 300 nm unless otherwise noted. TLC was carried out on Kieselgel 60 F_254_ plates (Merck, Germany) in the proper solvent systems (see below) and visualized by UV irradiation, ninhydrin (amine groups) or cystein/aqueous sulfuric acid (nucleoside and trityl groups). Preparative silica gel column chromatography, RPC, and cation exchange chromatography were performed using silica gel (35–70 μm, Acros Organics, USA), Porasil C 18 (55–105 μm, 125 A) (Waters, USA), and, Servacel P-23 (Serva, Germany) or SP Sepharose fast flow (GE Healthcare, USA), respectively. The compositions of all liquid mixtures are indicated as v/v percent. All evaporations were performed under reduced pressure. Compounds **1a,d** and **3a,d** were synthesized according to the published procedures [8,19].

### Synthesis of thymine containing monomers **1e** and **3e**

**{2-[*N*-(*tert*-Butyloxycarbonyl)aminomethyl]-6-(thymin-1-yl)morpholin-4-yl}acetic acid (1e):** Thymine containing monomer **1e** was synthesized similarly to uracil analogue **1d** [19] starting from 5-methyluridine (1.29 g, 5 mmol). Yield 1.0 g (2.50 mmol, 50%). *R*_f_ 0.68 (iPrOH/H_2_O, 4:1); ^1^H NMR (DMSO-*d*_6_) 7.53 (s, 1H, *H6*-Thy), 6.95 (t, *J* = 5.6 Hz, 1H, Boc-*NH*), 5.58 (dd, *J* = 10.1, 1.8 Hz, 1H, *H6*), 3.74–3.64 (m, 1H, *H2*), 3.11–2.74 (m, 6H, *H3*, *H5*, Boc-HN*CH_2_*, *CH_2_*C(O)OH), 2.30 (app. t, *J* = 10.3 Hz, 1H, *H3*), 2.01 (app. t, *J* = 10.6 Hz, 1H, *H5*), 1.77 (s, 3H, *СН_3_*-Thy), 1.37 (s, 9H, *CH_3_*-Boc); MALDI–TOFMS (*m*/*z*): [M + H]^+^ calcd for C_17_H_27_N_4_O_7_, 399.19; found, 399.77; [M + Na]^+^ calcd for C_17_H_26_N_4_NaO_7_, 421.17; found, 421.64. See Supporting Information File 1 for the synthetic scheme and physicochemical data of intermediate compounds.

**2-Aminomethyl-4-trityl-6-(thymin-1-yl)morpholine (3e):** Thymine containing monomer **3e** was synthesized similarly to uracil analogue **3d** [8] starting from the corresponding 2-hydromethyl derivative (2.16 g, 4.24 mmol). Yield 1.05 g, (2.20 mmol, 52%). *R*_f_ 0.12 (EtOH/DCM, 1:9); ^1^H NMR (CDCl_3_) 7.50–7.38 (br.s, 6H, *o-H*-Tr), 7.28 (app.t, *J* = 7.5 Hz, 6H, *m*-*H*-Tr), 7.17 (t, *J* = 7.2 Hz, 3H, *p*-*H*-Tr), 6.98 (q, *J* = 0.8 Hz, 1H, *H6*-Thy), 6.15–6.07 (dd, *J* = 9.8 Hz, 1H, 2.2, *H6*), 4.14–4.04 (m, 1H, *H2*), 3.31 (dt, *J* = 11.2, 2.3 Hz, 1H, *H3*), 3.05 (dt, *J* = 11.8, 2.1 Hz, 1H, *H5*), 2.75–2.64 (m, 2H, NH_2_*CH_2_*), 1.81 (d, *J* = 0.8 Hz, 3H, *CH_3_*), 1.39 (dd, *J* = 11.1, 10.0 Hz, 1H, *H3*), 1.35–1.30 (dd, *J* = 11.6, 10.6 Hz, 1H, *H5*); MALDI–TOFMS (*m*/*z*): [M – Tr + 2H]^+^ (the Tr protective group was removed during the sample preparation) calcd for C_10_H_17_N_4_O_3_ 241.13; found, 241.45. Physicochemical characteristics for intermediate compounds see in Supporting Information File 1.

#### General procedure for the synthesis of Boc-protected 2-aminomethylmorpholino monomers **5a,d,e**

**2-[*N*-(*tert*-Butyloxycarbonyl)aminomethyl]-6-(*N*^6^-benzoyladenin-9-yl)morpholine (2a), 2-[*N*-(*tert*-butyloxycarbonyl)aminomethyl]-6-(uracil-1-yl)morpholine (2d), 2-[*N*-(*tert*-butyloxycarbonyl)aminomethyl]-6-(thymin-1-yl)morpholine (2e):** The morpholino nucleoside **3a,d,e** (0.72 mmol), TEA (0.35 mL, 2.1 mmol), and (Boc)_2_O (0.19 g, 0.87 mmol) in pyridine (1.5 mL) were stirred for 1 h. The reaction mixture was diluted with DCM (30 mL) and washed with 5% aqueous NaHCO_3_ (2 × 25 mL). The organic layer was evaporated with toluene several times to remove traces of pyridine. The residue was dissolved in 80% aqueous acetic acid (AcOH) (5 mL). After 30 min of stirring, the mixture was poured over ice (50 g) and carefully neutralized by adding the calculated amount of dry NaHCO_3_ (5.6 g) under vigorous stirring. The aqueous suspension was then washed with diethyl ether (50 mL). The aqueous layer was concentrated to 10 mL; the target product was purified by RPC in the gradient of EtOH in water (0–60%). The appropriate fractions were evaporated to give after drying the title compounds (0.36 mmol, yield 50%).

Compound **2a** *R*_f_ 0.14 (EtOH/DCM, 1:9), 0.52 (iPrOH/H_2_O, 4:1); ^1^H NMR (CDCl_3_) 9.01 (s, 1H, Bz-*NH*), 8.78 (s, 1H, *H8*-Ade), 8.17 (s, 1H, *H2*-Ade), 8.01 (dt, *J* = 7.6, 1.2 Hz, 2H, *o*-*H*-Bz), 7.60 (tt, *J* = 7.5, 1.2 Hz, 1H, *p*-*H-*Bz), 7.50 (app. t, *J* = 7.5 Hz, 2H, *m*-*H-*Bz), 5.91 (dd, *J* = 10.1, 2.7 Hz, 1H, *H6*), 4.85 (t, *J* = 4.6 Hz, 1H, Boc-*HN*CH_2_), 3.9–3.86 (m, 1H, *H2*), 3.47–3.37 (m, 1H, Boc-HN*CH_2_*), 3.31 (dd, *J* = 12.2, 2.4 Hz, 1H, *H3*), 3.20–3.11 (m, 1H, Boc-HN*CH_2_*), 3.08 (dd, *J* = 10.5, 12.1 Hz, 1H, *H5*), 3.00 (dd, *J* = 12.6, 1.9 Hz, 1H, *H3*), 2.70 (dd, *J* = 12.6, 11.0 Hz, 1H, *H5*), 1.41 (s, 9H, *CH_3_*); MALDI–TOFMS (*m*/*z*): [M + H]^+^ calcd for C_22_H_28_N_7_O_4_, 454.22; found, 454.62; [M + Na]^+^ calcd for C_22_H_27_N_7_NaO_4_, 476.20; found, 476.68.

Compound **2d**: *R*_f_ 0.12 (EtOH/DCM, 1:9), 0.53 (iPrOH/H_2_O, 4:1); ^1^H NMR (CD_3_OD) 7.76 (d, *J* = 8.0 Hz, 1H, *H6*-Ura), 5.69 (d, *J* = 8.0 Hz, 1H, *H5*-Ura), 5.67 (dd, *J* = 10.2, 2.3 Hz, 1H, *H6*), 3.86–3.77 (m, 1Н, *H2*), 3.20 (br.d, *J* = 5.7 Hz, 2H, Boc-NH*CH_2_*), 3.04 (dd, *J* = 12.6, 2.3, 1H, *H3*), 2.88 (dd, *J* = 13.0, 2.0 Hz, 1H, *H5*), 2.64 (dd, *J* = 12.6, 10.4 Hz, 1H, *H3*), 2.51 (dd, *J* = 13.0, 11.0 Hz, 1H, *H5*), 1.43 (s, 9H, *CH_3_*); MALDI–TOFMS (*m*/*z*): [M + H]^+^ calcd for C_14_H_23_N_4_O_5_, 327.17; found, 327.02; [M + Na]^+^ calcd for C_14_H_22_N_4_NaO_5_, 349.15; found, 349.02.

Compound **2e**: *R*_f_ 0.19 (EtOH/DCM, 1:9), 0.77 (iPrOH/H_2_O, 4:1); ^1^H NMR (CDCl_3_) 7.21 (q, *J* = 1.1 Hz, 1H, *H6*-Thy), 5.67 (dd, *J* = 10.0, 2.6 Hz, 1H, *H6*), 4.84 (t, *J* = 5.2 Hz, 1H, Boc-*NH*CH_2_), 3.82–3.74 (m, 1H, *H2*), 3.44–3.35 (m, 1H, Boc-NH*CH_2_*), 3.13–3.02 (m, 2Н, Boc-NH*CH_2_*, *H3*), 2.91 (dd, *J* = 12.8, 1.7 Hz, 1H, *H5*), 2.61 (dd, *J* = 10.2, 12.2 Hz, 1H, *H3*), 2.52 (dd, *J* = 12.8, 11.3 Hz, 1H, *H5*), 1.92 (d, *J* = 1.1 Hz, 3H, *CH_3_*-Thy), 1.42 (s, 9H, *CH_3_*-Boc); MALDI–TOFMS (*m*/*z*): [M + H]^+^ calcd 341.18 for C_15_H_25_N_4_O_5_, found, 340.94; [M + Na]^+^ calcd for C_15_H_24_N_4_NaO_5_, 363.16; found, 362.96.

**6-[*N*-(2-methoxy-2-oxoacetyl)]aminohexanoic acid (4):** A mixture of dimethyl oxalate (1.20 g, 10 mmol), 6-aminohexanoic acid (1.31 g, 10 mmol) and TEA (10 mmol, 1.40 mL) in dry MeOH (20 mL) was stirred at room temperature for 24 h. The reaction mixture was evaporated; the residue was triturated with diethyl ether (25 mL) and cooled in an ice bath. Diethyl ether was decanted, and the residue was dried in vacuum to give the title compound **4** as a partial TEA salt (2.62 g, 8.2 mmol, colourless semisolid at room temperature). ^1^H NMR (DMSO-*d*_6_) 8.92 (t, *J* = 5.8 Hz, 1H, *NH*), 3.76 (s, 3H, *OCH_3_*), 3.10 (app. q, *J* = 6.7 Hz, 2H, NH*CH_2_*CH_2_), 2.65 (q, *J* = 7.2 Hz, 2H, N*CH_2_*CH_3_), 2.17 (t, *J* = 7.4 Hz, 2H, CH_2_*CH_2_*C(O)OH), 1.54–1.38 (m, 4H, NHCH_2_*CH_2_*CH_2_, CH_2_*CH_2_*CH_2_C(O)OH), 1.30–1.18 (m, 2H, CH_2_CH_2_*CH_2_*CH_2_CH_2_), 1.01 (t, *J* = 7.2 Hz, 3H, NCH_2_*CH_3_*); ^13^C NMR (DMSO-*d*_6_) 175.0, 161.6, 157.1, 53.1, 45.8, 39.1, 34.2, 28.6, 26.2, 24.6, 10.6.

**9-{2-[*N*-(*tert*-Butyloxycarbonyl)aminomethyl]-6-(*N^6^*-benzoyladenin-9-yl)morpholin-4-yl}-8,9-dioxo-7-azanonanoic acid (5a), 9-{2-[*N*-(*tert*-butyloxycarbonyl)aminomethyl]-6-(uracil-1-yl)morpholin-4-yl}-8,9-dioxo-7-azanonanoic acid (5d), 9-{2-[*N*-(*tert*-butyloxycarbonyl)aminomethyl]-6-(thymin-1-yl)morpholin-4-yl}-8,9-dioxo-7-azanonanoic acid (5e):** Boc-protected morpholino nucleosides **3a,d,e** (0.35 mmol), the acid **4** (0.76 g, 3.5 mmol), and TEA (0.42 mL, 3 mmol) in pyridine (3 mL) were heated at 50 °C for 48 h. The reaction mixture was then cooled and diluted with DCM (30 mL). The solution was washed with water (30 mL). The aqueous layer was extracted with DCM (2 × 30 mL). The organic layers were combined and evaporated several times with water to remove pyridine. The target product was purified by RPC in the gradient of MeCN in water (0–50%). The appropriate fractions were evaporated to give after drying the title compounds as a light cream powder. Yield 0.28 mmol, 80%.

Compound **5a**: *R*_f_ 0.43 (EtOH/DCM, 1:9), 0.73 (iPrOH/H_2_O, 4:1); ^1^H NMR (CD_3_OD) 8.76, 8.75 (2s, 0.5H each, *H8*-Ade), 8.67, 8.66 (2s, 0.5H each, *H2*-Ade), 8.10 (br.dt, *J* = 8.4, 1.2, 2H, *o-H-*Bz), 7.68 (br.tt, *J* = 7.5, 1.2 Hz, 1H, *p*-*H-*Bz), 7.59 (br.tt, *J* = 7.8, 1.3 Hz, 2H, *m*-*H-*Bz), 6.09, 6.02 (2dd, *J* = 10.5, 2.8 Hz, 0.5H each, *H6*), 4.82–4.78 (br.d, *J* = 11.8 Hz, 0.5H, *H3*), 4.66–4.58 (m, 2.5H, *H5*, Boc-NH*CH_2_*), 4.52 (dt, *J* = 13.2, 2.2 Hz, 0.5H, *H3*), 4.21 (br.d, *J* = 13.2 Hz, 0.5H, *H5*), 4.05–3.93 (m, 1H, *H2*), 3.94 (dd, *J* = 12.6, 10.6 Hz, 0.5H, *H3*), 3.58 (dd, *J* = 12.2, 10.6 Hz, 0.5H, *H5*), 3.28–3.23 (m, 2H, NH*CH*_2_CH_2_-), 2.89 (dd, *J* = 13.5, 11.4 Hz, 0.5H, *H3*), 2.32 (t, *J* = 7.2 Hz, 1H, CH_2_*CH*_2_C(O)OH), 2.32–2.28 (m, 0.5H, *H5*), 2.23 (t, *J* = 7.2 Hz, 1H, CH_2_*CH*_2_C(O)OH), 1.72–1.55 (m, 4H, NHCH_2_*CH*_2_, *CH*_2_CH_2_C(O)OH), 1.44, 1.43 (2s, 4.5H each, *CH_3_*), 1.43–1.36 (m, 2H, *CH*_2_CH_2_CH_2_C(O)OH); MALDI–TOFMS (*m*/*z*): [M + H]^+^ calcd for C_30_H_39_N_8_O_8_, 639.29; found, 639.23; [M + Na]^+^ calcd for C_30_H_38_N_8_NaO_8_, 661.27; found, 661.22; [M + K]^+^ calcd for C_30_H_38_KN_8_O_8_, 677.24; found, 676.19.

Compound **5d**: *R*_f_ 0.45 (EtOH/DCM, 1:9), 0.79 (iPrOH/H_2_O, 4:1); ^1^H NMR (CD_3_OD) 7.83, 7.80 (2d, *J* = 8.2 Hz, 0.5H each, *H6*-Ura), 5.76, 5.74 (2d, *J* = 8.2 Hz, 0.5H each, *H5*-Ura), 5.74, 5.71 (2dd, *J* = 10.2, 2.9 Hz, 0.5 each, *H6*), 4.75–4.59 (m, 2H, Boc-NH*CH_2_*), 4.56 (br.d, *J* = 12.9 Hz, 0.5H, *H3*), 4.44 (dt, *J* = 13.1, 2.0 Hz, 0.5H, *H5*), 4.26 (br.d, *J* = 12.9 Hz, 0.5H, *H3*), 4.10 (dt, *J* = 13.1, 1.8 Hz, 0.5H, *H5*), 3.91–3.81 (m, 1H, *H2*), 3.33–3.28 (m, 2.5H, *H3,* NH*CH*_2_CH_2_), 3.14 (dd, *J* = 13.4, 11.5 Hz, 0.5H, *H5*), 2.94 (dd, *J* = 13.2, 10.4 Hz, 0.5H, *H3*), 2.76 (dd, *J* = 13.3, 11.4 Hz, 0.5H, *H5*), 2.33, 2.31 (2t, *J* = 7.5 Hz, 2H, CH_2_*CH*_2_C(O)OH), 1.72–1.56 (m, 4H, NHCH_2_*CH*_2_, *CH*_2_CH_2_C(O)OH), 1.47, 1.46 (2s, 4.5H each, *CH_3_*), 1.47–1.37 (m, 2H, *CH*_2_CH_2_CH_2_C(O)OH); MALDI–TOFMS (*m*/*z*): [M + H]^+^ calcd for C_22_H_34_N_5_O_9_, 512.24; found, 512.07; [M + Na]^+^ calcd for C_22_H_33_N_5_NaO_9_, 534.22; found, 534.20; [M + K]^+^ calcd for C_22_H_33_KN_5_O_9_, 550.19; found, 550.16.

Compound **5e**: *R*_f_ 0.68 (EtOH/DCM, 1:9), 0.83 (iPrOH/H_2_O, 4:1); ^1^H NMR (CD_3_OD) 7.67, 7.64 (2q, *J* = 1.1 Hz, 0.5 each, *H6*-Thy), 5.74, 5.70 (2dd, *J* = 10.0, 2.7 Hz, 0.5 each, *H6*), 4.75–4.58 (m, 2H, Boc-NH*CH_2_*), 4.51 (br.d, *J* = 12.9 Hz, 0.5H, *H3*), 4.43 (dt, *J* = 13.6, 2.0 Hz, 0.5H, *H5*), 4.21 (br.d, *J* = 12.8 Hz, 0.5H, *H3*), 4.09 (dt, *J* = 13.1, 2.2 Hz, 0.5H, *H5*), 3.89–3.80 (m, 1H, *H2*), 3.32–3.26 (m, 2.5H, *H3,* NH*CH*_2_CH_2_), 3.15 (dd, *J* = 13.6, 11.4 Hz, 0.5H, *H5*), 2.99 (dd, *J* = 13.1, 10.5 Hz 0.5H, *H3*), 2.76 (dd, *J* = 13.1, 11.4 Hz, 0.5H, *H5*), 2.34, 2.32 (2t, *J* = 7.5 Hz*,* 2H, CH_2_*CH*_2_C(O)OH), 1.93, 1.92 (2d, *J* = 1.1 Hz, 1.5H each, *CH_3_*-Thy), 1.71–1.56 (m, 4H, NHCH_2_*CH*_2_, *CH*_2_CH_2_C(O)OH), 1.47, 1.46 (2s, 4.5H each, *CH_3_-*Boc), 1.47–1.37 (m, 2H, *CH*_2_CH_2_CH_2_C(O)OH); MALDI–TOFMS (*m*/*z*): [M + Na]^+^ calcd for C_23_H_35_N_5_NaO_9_, 548.23; found, 548.27; [M + K]^+^ calcd for C_23_H_35_KN_5_O_9_, 564.21; found, 564.26

#### Loading of the support with the first monomer

Boc-Gly-PAM resin (10–30 mg) was placed in the receiver column equipped with a cap at the outlet. Loading of the resin was performed according the protocol (Table 1) using the monomers **5a,d,e** for the coupling step. The column was shaken to mix resin and reactants. Changing the reactants and solvents was carried out by centrifugation of the column equipped with a collection tube at 2000 min^−1^. After drying, a sample of the resin (1–2 mg) was treated with a mixture of iPrOH and concentrated (25%) aqueous ammonia (0.1 mL, 1:1) for 48 h at room temperature under shaking. The substitution of the supports **6a,d,e** was determined after calculating the amount of the cleaved monomer according the data of quantitative analytical HPLC using the extinction coefficients ε_260_ 15.02 mM^−1^cm^−1^ for A, 9.66 for U and 8.56 for T [26] and was found to be 0.60–0.65 mmol/g. Physicochemical characteristics of the cleaved monomers are given in Supporting Information File 1.

#### Synthesis of MorGly homopentamers **7a,d,e**

Pentamers **7a,d,e** were synthesized manually in the receiver columns (see above) on the supports **6a,d,e** (10 mg), respectively, by repeating steps 2–8 of the protocol (Table 1). Monomers **1a,d,e** were used for the coupling step. Physicochemical characteristics of oligomers cleaved from the support after certain cycles are given in Supporting Information File 1. After completion of the synthesis, the oligomers were cleaved from the support by concentrated (25%) aqueous ammonia/iPrOH (1:1, 1 mL) treatment for 48 h at room temperature under shaking. The supernatants were evaporated. The Boc-protected oligomers were treated with TFA (0.5 mL) for 5 min and evaporated. Crude deprotected oligomers were dissolved in water containing 0.1% TFA (1 mL) and subjected to cation exchange chromatography (SP Sepharose) in a gradient of NaCl (0–2M) in aqueous 20% ethanol containing 0.1% TFA. The appropriate fractions were evaporated, and the target pentamers were purified by RPC in the gradient of MeCN in water (0–40%) containing 0.1% TFA. The appropriate fractions were concentrated using vacuum centrifugation. After purification, 0.5–1.0 μmol of homopentamers **7a,d,e** were obtained. RPC HPLC traces of purified pentamers **7a,d,e** are provided in Supporting Information File 1.

Pentamer **7a**: MALDI–TOFMS: (*m*/*z*): [M + H]^+^ calcd for C_58_H_76_N_36_O_9_, 1406.66; found, 1406.43.

Pentamer **7d**: MALDI–TOFMS (*m*/*z*): [M + H]^+^ calcd for C_53_H_71_N_20_O_19_, 1291.52; found, 1291.65.

Pentamer **7e**: MALDI–TOFMS (*m*/*z*): [M + H]^+^ calcd for C_58_H_81_N_20_O_19_, 1361.60; found, 1361.71.

## Supporting Information

Synthetic schemes, procedures and physicochemical data for intermediates in the synthesis of compounds **1e** and **3e**; kinetics of the cleavage of monomers from supports **6a,d**; TLC, RPC HPLC traces and mass spectra data for oligomers cleaved from the samples of supports **6a,d,e** and for purified pentamers **7a,d,e** are provided in the Supporting Information.

File 1 Syntheses and characteristics for selected compounds.
