# Supplementary material for: Solid-phase-supported synthesis of morpholinoglycine oligonucleotide mimics
Source: Beilstein J Org Chem. 2014 May 20;10:1151–8. doi: 10.3762/bjoc.10.115 (PMC4077379; doi:10.3762/bjoc.10.115)
Supplement: File 1 — Syntheses and characteristics for selected compounds. [file Beilstein_J_Org_Chem-10-1151-s001.pdf]

Solid-phase-supported synthesis of morpholino-glycine oligonucleotide mimics

Tatyana V. Abramova[*1], Sergey S. Belov[1,2], Yulia V. Tarasenko[1] and Vladimir N. Silnikov[1]

Address: [1]Institute of Chemical Biology and Fundamental Medicine, SB RAS, Lavrent'ev Ave, 8, Novosibirsk 630090, Russia, [2]Novosibirsk State University, Pirogova St. 2, Novosibirsk 630090, Russia

Email: Tatyana V. Abramova[*] - abramova@niboch.nsc.ru

* Corresponding author

Syntheses and characteristics for selected compounds.

Synthetic scheme and physicochemical data for intermediates in the synthesis of compound 1e

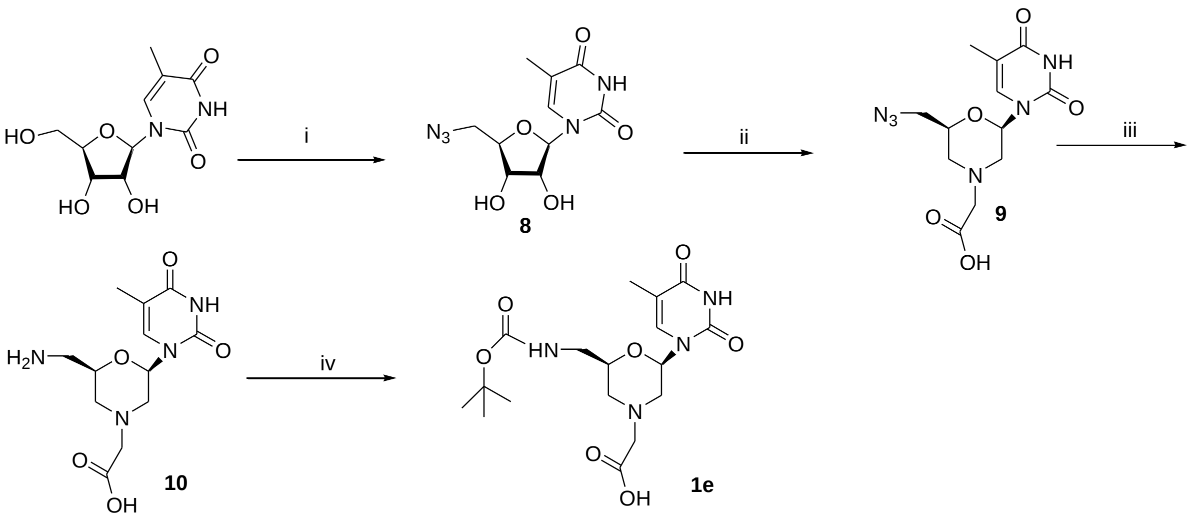

Scheme S1: Synthesis of compound **1e**: i) PPh_3, NaN_3, $CBrCl_3$, DMF; ii) $NaIO_4$, TEA, Gly, then $NaCNBH_3$; iii) PPh_3, Py, then NH_3/H_2O; iv) 1 M NaOH, iPrOH, $(Boc)_2O$;

5'-Azido-5-methyluridine (8)

5-Methyluridine (1.29 g, 5 mmol), triphenylphosphine (2.89 g, 11 mmol), NaN_3 (3.26 g, 50 mmol), and $CBrCl_3$ (1.1 mL, 11 mmol) were stirred in DMF (50 mL) overnight. The reaction mixture was then evaporated, the residue was suspended in DCM (50 mL), and the suspension was washed with water (50 mL). The aqueous layer was evaporated; the residue was dissolved in a minimal amount of EtOH (ca. 5 mL) and placed on a column with the reversed phase equilibrated with water. The target product was purified by RPC in a gradient of EtOH in water (0–30 %). The appropriate fractions were evaporated affording the title compound as a light cream powder after drying. Yield was 1.13 g, 4.0 mmol, 80%. R_f = 0.61 (EtOH/DCM, 1/9); IR (KBr): 2200 cm^{-1} (N≡N); ^1H NMR (DMSO-d_6): 11.35 (s, 1H, *NH*), 7.49 (d, 1H, *J* = 0.8, *H6*), 5.76 (d, 1H, *J* = 5.8, *H1'*), 4.13 (app. t, 1H, *J* = 5.5, *H4'*), 3.94-3.87 (m, 2H, *H2', H3'*), 3.57 (d, 2H, *J* = 4.8, *H5', H5'*), 1.78 (q, 3H, *J* = 0.8, *CH_3*).

2-Azidomethyl-6-(thymine-1-yl)morpholin-4-yl acetic acid (9)

$NaIO_4$ (0.95 g, 4.4 mmol) was dissolved in warm water (10 mL) and added to the solution of nucleoside **8** (1.13 g, 4.0 mmol) in EtOH (80 mL). After 20 min of stirring, glycine (0.33 g, 4.4 mmol) in a mixture of water (25 mL) and TEA (0.53 mL) was added to the above suspension. The reaction mixture was stirred for 2 h; pH was maintained near 9.0–9.5 by adding TEA (about 0.3 mL) in portions. The precipitate was then removed by filtration, and $NaCNBH_4$ (0.4 g, 6.3 mmol) was added to the filtrate. After stirring for 30 min, the reaction mixture was acidified to pH 4.0 by adding TFA (about 1.5 mL). After stirring for 3 h, the reaction mixture was evaporated. The target product was purified by RPC in a gradient of EtOH in water (0–30 %) containing 0.1 %TFA. The appropriate fractions were evaporated affording the title compound as a glass-like residue after drying. Yield was 1.16 g, 3.6 mmol, 80%. R_f = 0.73 (iPrOH/H_2O, 4:1); IR (KBr): 2100 cm^{-1} (N≡N); ^1H NMR (DMSO-d6) 11.46 (s, 1H, *NH*-Thy), 7.47 (q, 1H, *J* = 1.0, *H6*-Thy), 5.82 (dd, 1H, *J* = 10.4, 2.1, *H6*), 4.12-4.04 (m, 1H, *H2*), 3.67-3.57 (m, 3H, CH_2C(O)OH, N_3CH_2), 3.40-3.33 (m, 1H, N_3CH_2), 3.19-3.03 (m, 2H, *H3, H5*), 2.82 (app.t, 1H, *J* = 10.6, *H3*), 2.62 (app.t, 1H, *J* = 11.9, *H5*), 1,76 (d, 3H, *J* = 1.0, CH_3).

2-Aminomethyl-6-(thymine-1-yl)morpholin-4-yl acetic acid (10)

Morpholino nucleoside **9** (1.16 g, 3.6 mmol) was dissolved in Py (6 mL), and triphenylphosphine (1.02 g, 3.8 mmol) was added to the solution. The reaction mixture was stirred overnight, followed by the addition of concentrated (25%) aqueous ammonia (6 mL). The reaction mixture was stirred for 8 h and evaporated several times with water to remove Py. The residue was suspended in 0.1% aqueous TFA (100 mL); the suspension was filtered, and the filtrate was applied to a column with Servacel P-23 in an H^+ form. The target product was purified by cation exchange

chromatography in a linear gradient of NH_4HCO_3 (0–0.2 M) in aqueous 20% EtOH. The appropriate fractions were evaporated. Evaporation was repeated several times with EtOH to remove traces of buffer. The title compound was obtained as a cream powder after drying. Yield was 0.93 g, 3.10 mmol, 86%. R_f = 0.56 (iPrOH/conc. aq. ammonia/H_2O, 7/1/2); ^{1}H NMR (DMSO-d_6) 7.63 (q, 1H, *J* = 0.9, *H6*-Thy), 5.60 (dd, 1H, *J* = 10.1, 2.5, *H6*), 3.85-3.78 (m, 1H, *H2*), 3.05-2.77 (m, 6H, *H3, H5,* H_2NCH_2, $CH_2C(O)OH$), 2.36 (app.t, 1H, *J* = 11.5, *H3*), 2.12 (t, 1H, *J* = 11.1, *H5*), 1.77 (d, 3H, *J* = 0.9, CH_3).

2-[*N*-(*tert*-Butyloxycarbonyl)aminomethyl]-6-(thymin-1-yl)morpholin-4-yl acetic acid (1e)

Morpholino nucleoside 10 (0.93 g, 3.10 mmol) was dissolved in a mixture of 1 M NaOH (3.1 mL) and iPrOH (3.1 mL). Di-*tert*-butyl dicarbonate (1.6 mL, 7 mmol) was added. The mixture was heated at 40 °C to start the reaction and then stirred at room temperature for 2 h. The solution was then extracted with diethyl ether (2 × 6 mL); the aqueous layer was concentrated twofold, diluted with water (20 mL), and acidified to pH 4.5 with a citric acid solution. The target product was purified by RPC in a linear gradient of EtOH in water (0–50%). The appropriate fractions were evaporated affording the title compound as a light cream powder after drying. Yield and physicochemical data of **1e** are given in the Experimental part of the article.

Synthetic scheme and physicochemical data for intermediates in the synthesis of compound 3e

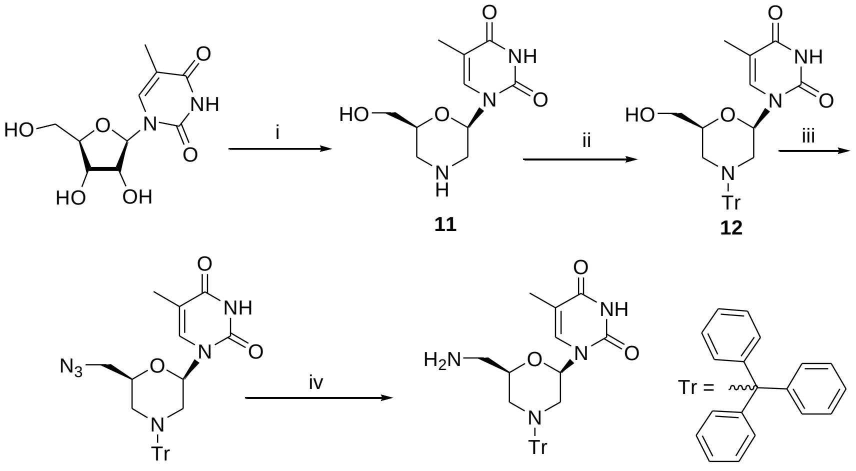

Scheme S2: Synthesis of compound **3e**: i) $NaIO_4$ $(NH_4)_2B_4O_7 \times 4H_2O$, TEA, then $NaCNBH_3$; ii) TrCl, TEA, DMF; iii) PPh_3, NaN_3, $CBrCl_3$, DMF; iv) PPh_3, 1M NaOH, Py.

2-Hydroxymethyl-4-trityl-6-(thymin-1-yl)morpholine (12)

$NaIO_4$ (2.25 g, 10.5 mmol) was dissolved in warm water (10 mL) and added to the solution of 5-methyluridine (2.58 g, 10 mmol) in EtOH (200 mL). After 10 min of stirring, $(NH_4)_2B_4O_7 \times 4H_2O$ (3.16 g, 12 mmol) was added. The suspension was stirred for 2 h while maintaining pH at 8.5–9.0 by adding TEA (about 3.3 mL). The precipitate was removed by filtration; and $NaCNBH_4$ (0.83 g, 12 mmol) was added to the filtrate. After stirring for 1 h, the reaction mixture was acidified to pH 4.0 by adding TFA (about 3–5 mL). The reaction mixture was stirred for 3 h, followed by evaporation. Evaporation was repeated with water (2 × 50 mL) and a mixture of water and TEA (9:1, 50 mL). The crude product **11** was subjected to the next step without purification.

The residue obtained at previous step was dried by coevaporation with CH_3CN (2 × 25 mL) and was suspended in DMF (50 mL). TEA (2.8 mL, 20 mmol) and

triphenylmethyl chloride (1.87 g, 6.7 mmol) were added to the reaction mixture. The suspension was stirred for 1 h while maintaining pH at 8.5–9.0 by adding TEA (about 1.5 mL). The reaction mixture was then poured into water (900 mL) under vigorous stirring. After 1 h, the precipitate was separated by filtration and dried. The resulting white powder was suspended in DCM (50 mL) and petroleum ether (40–70 °C, 900 mL) under vigorous stirring. After 30 min, the precipitate was separated by filtration and dried. Yield 2.72 g, 5.63 mmol, 56.3%. R_f 0.74 (EtOH/DCM, 1/9); ^{1}H NMR ($CDCl_3$) 8.27 (s, 1H, *NH*), 7.45 (d, 6H, *J* = 7.5, *o-H*-Tr), 7.28 (app.t, 6H, *J* = 7.7, *m-H*-Tr), 7.17 (t, 3H, *J* = 7.1, *p-H*-Tr), 6.97 (q, 1H, *J* 1.1, *H6*-Thy), 6.15 (dd, 1H, *J* = 9.7, 2.2, *H6*), 4.30-4.20 (m, 1H, *H2*), 3.65-3.51 (m, 2H, HOCH_2), 3.32 (dt, 1H, *J* = 11.4, 1.9, *H3*), 3.17-3.07 (dt, 1H, *J* = 12.0, 2.1, *H5*), 1.81 (d, 3H, *J* = 1.1, CH_3), 1.49-1.37 (m, 2H, *H3, H5*).

2-Azidomethyl-4-trityl-6-(thymin-1-yl)morpholine (13)

Morpholino nucleoside **12** (0.74 g, 1.53 mmol), triphenylphosphine (0.88 g, 3.37 mmol), NaN_3 (0.99 g, 15.3 mmol), and $CBrCl_3$ (0.34 mL, 3.37 mmol) were stirred in DMF (50 mL) overnight. The reaction mixture was then evaporated; the residue was suspended in DCM (50 mL); and the suspension was washed with water (50 mL). The organic layer was dried (Na_2SO_4) and evaporated. The target product was purified by silica gel chromatography in a gradient of acetone in DCM (0–25%). The appropriate fractions were evaporated affording the title compound as white foam after drying. Yield 0.62 g, 1.22 mmol, 80%. R_f 0.40 (EtOH/DCM, 0.5/9.5); IR (KBr): 2100 cm^{-1} (N≡N); ^{1}H NMR ($CDCl_3$): 8.23 (s, 1H, *NH*), 7.45 (6H, d, *J* = 6.5, *o-H*-Tr), 7.28 (app.t, 6H, *J* = 7.5, *m- H*-Tr), 7.18 (t, 3H, *J* = 7.2, *p- H*-Tr), 7.01 (q, 1H, *J* = 1.0, *H6*-Thy), 6.13 (dd, 1H, *J* = 9.6, 2.3, *H6*), 4.34-4.26 (m, 1H, *H2*), 3.39-3.31 (m, 2H, *H3,*

CH_2N_3), 3.24-3.17 (m, 1H, CH_2N_3), 3.06 (dt, 1H, *J* = 12.0, 2.2, *H5*), 1.81 (d, 3H, *J* = 1.0, CH_3), 1.53 (dd, 1H, *J* = 11.5, 10.7, *H3*), 1.41 (dd, 1H, *J* = 11.5, 9.9, *H5*).

2-Aminomethyl-4-trityl-6-(thymin-1-yl)morpholine (3e)

Morpholino nucleoside **13** (2.16 g, 4.24 mmol) was dissolved in Py (15 mL), and triphenylphosphine (1.16 g, 4.5 mmol) was added to the solution. The reaction mixture was stirred overnight, followed by the addition of aqueous 1 M NaOH (5 mL). After 6 h of stirring, the reaction mixture was diluted with DCM (150 mL) and washed with water (2x100 mL). The organic layer was dried (Na_2SO_4) and evaporated. The target product was purified by silica gel chromatography in a gradient of EtOH in DCM (0-50 %) containing TEA (0.1 %). The appropriate fractions were evaporated affording the title compound as white foam after drying. Yield and physicochemical data of 3e are given in the **Experimental part** of the article.

Kinetics of the cleavage of monomers from supports 6a,d

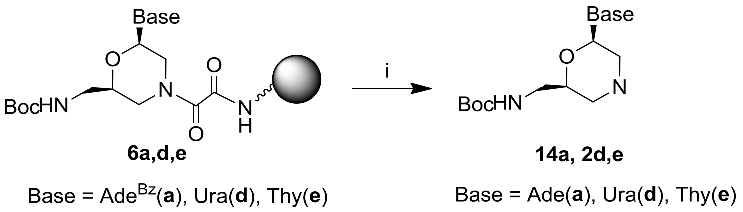

Scheme S3: Cleavage of the monomers **14a**, **2d**,**e** from the supports **6a,d,e,** respectively: i) NH_3/H_2O.

A sample of the support **6a,d,e** (1–2 mg) was placed in the capped tube and treated with a mixture of iPrOH/conc. aq. ammonia (1:1) at room temperature under shaking. Aliquots were periodically withdrawn, evaporated, dissolved in aqueous EtOH (50%), and analyzed on a Milichrom A02 chromatograph system (Econova, Russia) (see

Experimental part). The amount of products **14a** or **2d,e** released in the solution was calculated using the MultiChrom program package (Econova, Russia) and extinction coefficients are as specified in the Experimental part. Data for supports **6a** and **6d** are shown in the Figure S1.

A

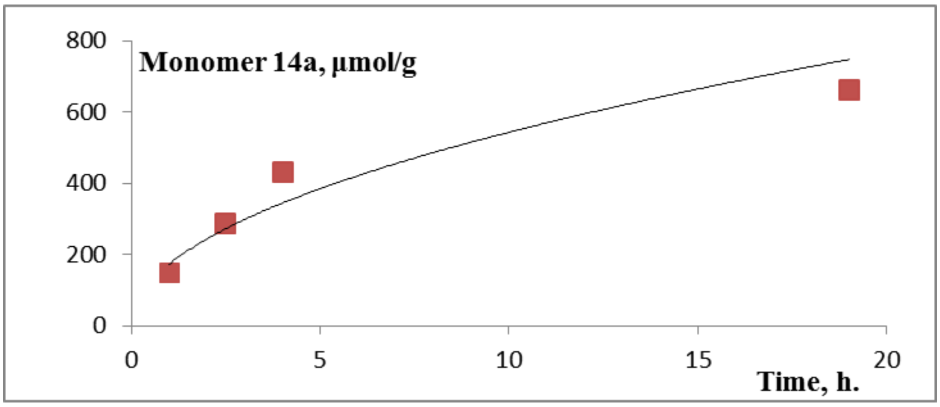

B

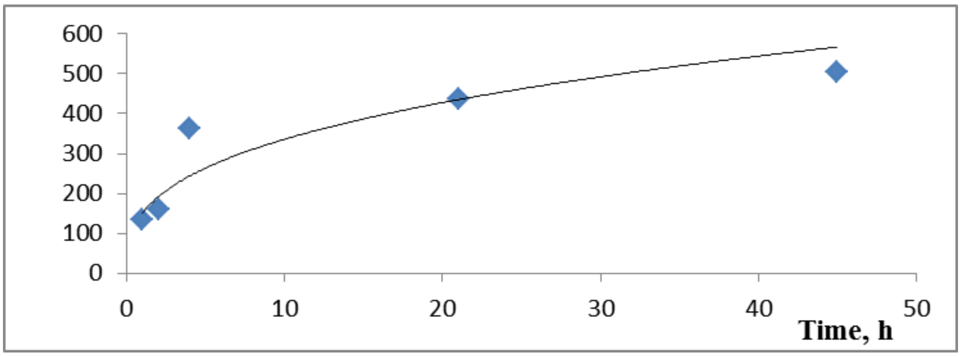

Figure S1: Release kinetics of monomers **14a** and **2d** from supports **6a** (**A**) and **6d** (**B**) during ammonia treatment, respectively.

TLC, RPC/HPLC traces and mass spectra data for oligomers cleaved from the samples of supports 6a,d,e

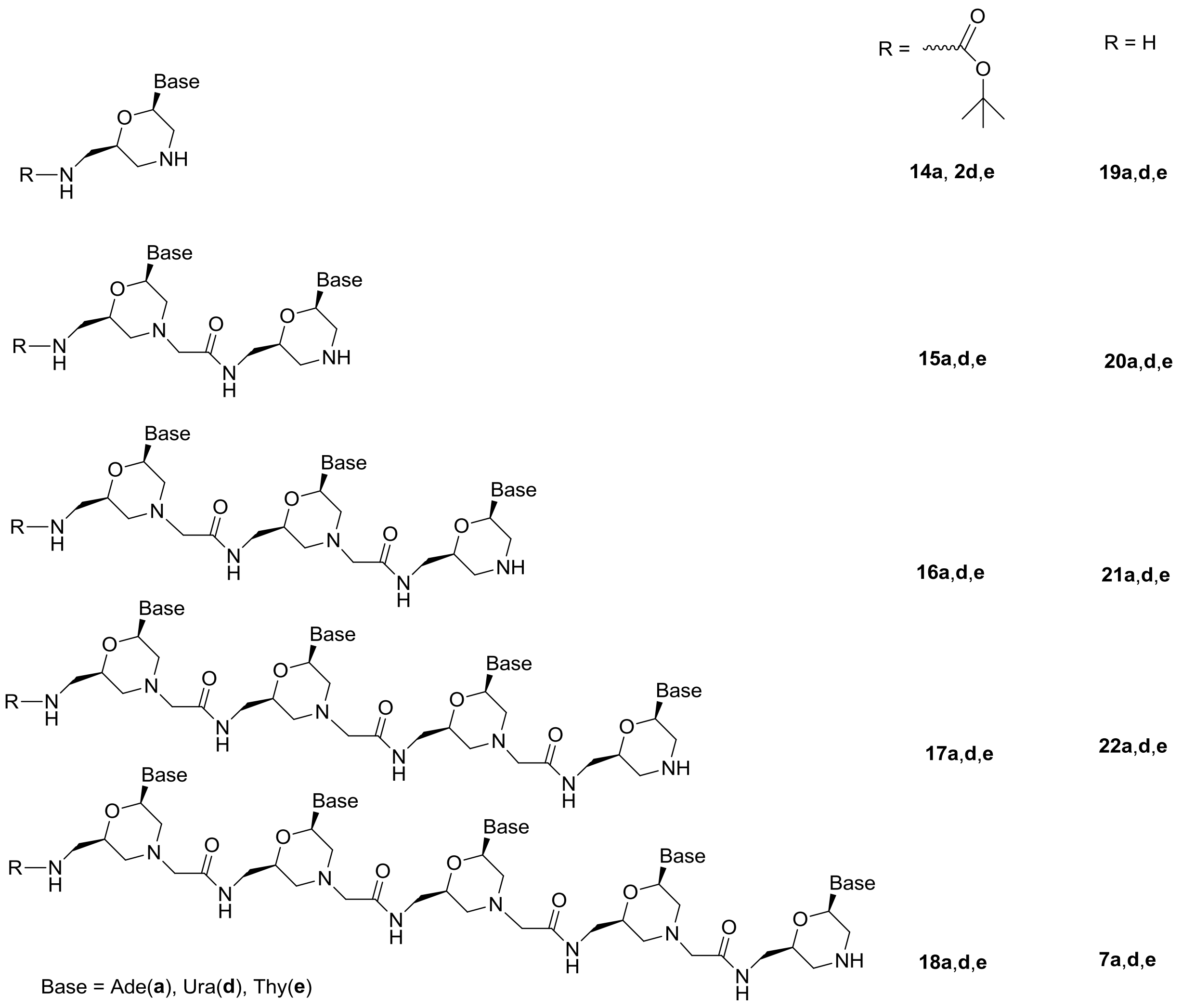

Figure S2: Oligomers cleaved from solid supports **6a,d,e** after certain synthesis cycles in the course of SPPS of homopentamers: after aqueous ammonia treatment (**2d,e; 14a,d,e–18a,d,e**) of the supports, and after subsequent TFA treatment of the products (**7a,d,e; 19a,d,e–22a,d,e**).

Table S1: TLC data for certain oligomers (Figure S2).

Base = Ade				Base = Ura				Base = Thy			
	R_f^a		R_f^b		R_f^a		R_f^b		R_f^a		R_f^b
**14a**	0.48	**19a**	n.d.	**2d**	0.53	**19d**	0.51	**2e**	0.77	**19e**	n.d.
**15a**	0.24	**20a**	n.d.	**15d**	0.43	**20d**	0.44	**15e**	0.55	**20e**	n.d.
**16a**	0.22	**21a**	n.d.	**16d**	0.36	**21d**	0.40	**16e**	0.40	**21e**	n.d.
**17a**	n.d.	**22a**	n.d.	**17d**	0.23	**22d**	0.36	**17e**	0.27	**22e**	n.d.
**18a**	0.11	**7a**	0.25	**18d**	0.13	**7d**	0.32	**18e**	0.16	**7e**	0.53

[a]iPrOH/H_2O, 4/1; [b]iPrOH/conc. aq. ammonia/H_2O, 7/1/2.

Table S2: MALDI–TOFMS data for Ade-containing oligomers (Figure S2).

	m/z
**14a**	$[M + H]^+$ calcd 350.19 for $C_{15}H_{24}N_7O_3$; found, 350.11; $[M + Na]^+$ calcd 372.18 for $C_{15}H_{23}N_7NaO_3$; found, 372.11;
**15a**	$[M + H]^+$ calcd 639.32 for $C_{27}H_{39}N_{14}O_5$; found, 639.22; $[M + Na]^+$ calcd 661.30 for $C_{27}H_{38}N_{14}NaO_5$; found, 661.20
**16a**	$[M + H]^+$ calcd 928.45 for $C_{39}H_{54}N_{21}O_7$; found, 928.48; $[M + Na]^+$ calcd 953.43 for $C_{39}H_{53}N_{21}NaO_7$; found, 950.46
**17a**	n.d.
**18a**	$[M + H]^+$ calcd 1506.71 for $C_{63}H_{84}N_{35}O_{11}$; found, 1506.96.
**19a**	n.d.
**20a**	n.d.
**21a**	n.d.
**22a**	n.d.
**7a**	$[M + H]^+$ calcd 1406.66 for $C_{58}H_{76}N_{35}O_9$; found, 1406.43; $[M + Na]^+$ calcd 1428.64 for $C_{58}H_{75}N_{35}NaO_9$; found, 1428.41.

Table 3: MALDI–TOFMS data for Ura-containing oligomers (Figure S2).

	m/z
**2d**	[M + H]$^+$ calcd 327.17 for $C_{14}H_{23}N_4O_5$; found, 327.02; [M + Na]$^+$ calcd 349.15 for $C_{14}H_{22}N_4NaO_5$; found, 349.02.
**15d**	[M + H]$^+$ calcd 593.27 for $C_{25}H_{37}N_8O_9$; found, 593.29; [M + Na]$^+$ calcd 615.25 for $C_{25}H_{36}N_8NaO_9$; found, 615.25; [M – Boc + 2H]$^+$ calcd 493.22 for $C_{20}H_{29}N_8O_7$; found, 493.19.
**16d**	[M + H]$^+$ calcd 859.37 for $C_{36}H_{51}N_{12}O_{13}$; found, 859.46; [M + Na]$^+$ calcd 881.35 for $C_{36}H_{50}N_{12}NaO_{13}$; found, 881.41; [M – Boc + 2H]$^+$ calcd 759.32 for C_{31}H43$N_{12}O_{11}$; found, 759.43.
**17d**	[M + H]$^+$ calcd 1125.47 for $C_{47}H_{65}N_{16}O_{17}$; found, 1125.43; [M + Na]$^+$ calcd 1147.45 for $C_{47}H_{64}N_{16}NaO_{17}$; found, 1147.40; [M – Boc + 2H]$^+$ calcd 1025.42 for $C_{42}H_{57}N_{16}O_{15}$; found, 1025.39.
**18d**	[M + H]$^+$ calcd 1391.57 for $C_{58}H_{79}N_{20}O_{21}$; found,1391.56; [M + Na]$^+$ calcd 1413.55 for $C_{58}H_{78}N_{20}NaO_{21}$; found,1413.56; [M – Boc + 2H]$^+$ calcd 1291.52 for $C_{53}H_{71}N_{20}O_{19}$; found, 1291.57.
**19d**	[M + H]$^+$ calcd 227.11 for $C_9H_{15}N_4O_3$; found, 226.92.
**20d**	[M + H]$^+$ calcd 493.22 for $C_{20}H_{29}N_8O_7$; found, 493.35; [M + Na]$^+$ calcd 515.20 for $C_{20}H_{28}N_8NaO_7$; found, 515.31.
**21d**	[M + H]$^+$ calcd 759.32 for $C_{31}H_{43}N_{12}O_{11}$; found, 759.30; [M + Na]$^+$ calcd 781.30 for $C_{31}H_{42}N_{12}NaO_{11}$; found, 781.29.
**22d**	[M + H]$^+$ calcd 1025.42 for $C_{42}H_{57}N_{16}O_{15}$; found, 1025.27; [M + Na]$^+$ calcd 1047.40 for $C_{42}H_{56}N_{16}NaO_{15}$; found, 1047.24.
**7d**	[M + H]$^+$ calcd 1291.25 for $C_{53}H_{71}N_{20}O_{19}$; found, 1291.65; [M + Na]$^+$ calcd 1313.50 for $C_{53}H_{70}N_{20}NaO_{19;}$ found, 1313.63.

Table S4: MALDI–TOFMS data for Thy-containing oligomers (Figure S2).

	m/z
**2e**	$[M + H]^+$ calcd 341.18 for $C_{15}H_{25}N_4O_5$; found, 340.94; $[M + Na]^+$ calcd 363.16 for $C_{15}H_{24}N_4NaO_5$; found, 362.96.
**15e**	$[M + H]^+$ calcd 621.30 for $C_{27}H_{41}N_8O_9$; found, 621.28; $[M + Na]^+$ calcd 643.28 for $C_{27}H_{40}N_8NaO_9$; found, 643.26.
**16e**	$[M + H]^+$ calcd 901.42 for $C_{39}H_{57}N_{12}O_{13}$; found, 901.41; $[M + Na]^+$ calcd 923.40 for $C_{39}H_{56}N_{12}NaO_{13}$; found, 923.40.
**17e**	$[M + H]^+$ calcd 1181.53 for $C_{51}H_{73}N_{16}O_{17}$; found, 1181.63; $[M + Na]^+$ calcd 1203.52 for $C_{51}H_{72}N_{16}NaO_{17}$; found, 1203.63.
**18e**	$[M + H]^+$ calcd 1461.65 for $C_{63}H_{89}N_{20}O_{21}$; found, 1461.76; $[M + Na]^+$ calcd 1483.63 for for $C_{63}H_{88}NaN_{20}O_{21;}$1 found, 483.77.
**19e**	n.d.
**20e**	n.d.
**21e**	n.d.
**22e**	n.d.
**7e**	$[M + H]^+$ calcd 1361.60 for $C_{58}H_{81}N_{20}O_{19}$; found, 1361.71; $[M + Na]^+$ calcd 1383.58 for $C_{58}H_{80}N_{20}NaO_{19}$; found, 1383.71.

RPC HPLC trace of the reaction mixture after ammonia treatment of the sample of the support 6a (main product – 14a)

Report date:	03.04.2014 13:32:24
Analysis from:	30.03.2012 11:19:57
File:	11190040.CHW
Vial number:	4
Volume:	5.0 µl
Dilution:	1.00
COLUMN:	ProntoSIL-120-5-C18 AQ DB-2003
Size:	2.0 x 75 mm
Part.size:	5.0 µm
ELUENT:	0% CH_3CN 0.1M TEAAc pH7.0
	80%CH_3CN 0.1M TEAAc pH7.0
Flow:	200.00 µl/min
Temperature:	35.0°C
Pressure:	6.1 MPa

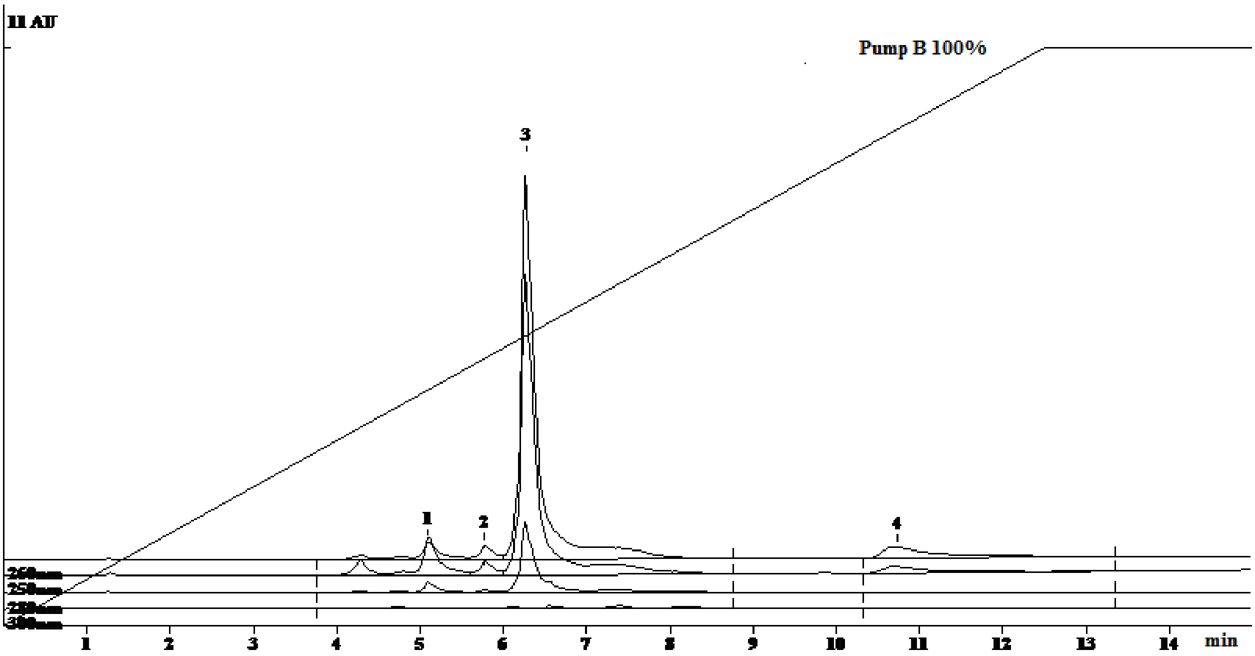

No	Retention µl	Height AU	Area AU*µl
1	1022.57	0.35	25.732
2	1157.64	0.28	10.516
3	1254.55	7.67	341.906
4	2142.45	0.22	35.983
4	2999.15	8.53	414.137

No	Retention µl	250nm	280nm (/260 nm)	300nm
1	1022.57	1.939	0.522	0.158
2	1157.64	0.990	0.252	0.037

3	1254.55	0.801	0.206	0.031
4	2142.45	0.565	0.003	-0.004

This report has been created by MultiChrom-Spectrum for Windows
Ampersand Ltd.

RPC HPLC trace of the reaction after ammonia treatment of the sample of the support 6a after 2 cycles with monomer 1a (main product – 16a)

Report date:	03.04.2014 13:35:20
Analysis from:	28.09.2012 12:44:33
File:	12440010.CHW
Vial number:	1
Volume:	5.0 µl
Dilution:	1.00
COLUMN:	ProntoSIL-120-5-C18 AQ DB-2003
Size:	2.0 x 75 mm
Part.size:	5.0 µm
ELUENT:	0% CH_3CN 0.1M TEAAc pH7.0
	80%CH_3CN 0.1M TEAAc pH7.0
Flow:	100.00 µl/min
Temperature:	35.0°C
Pressure:	5.1 MPa

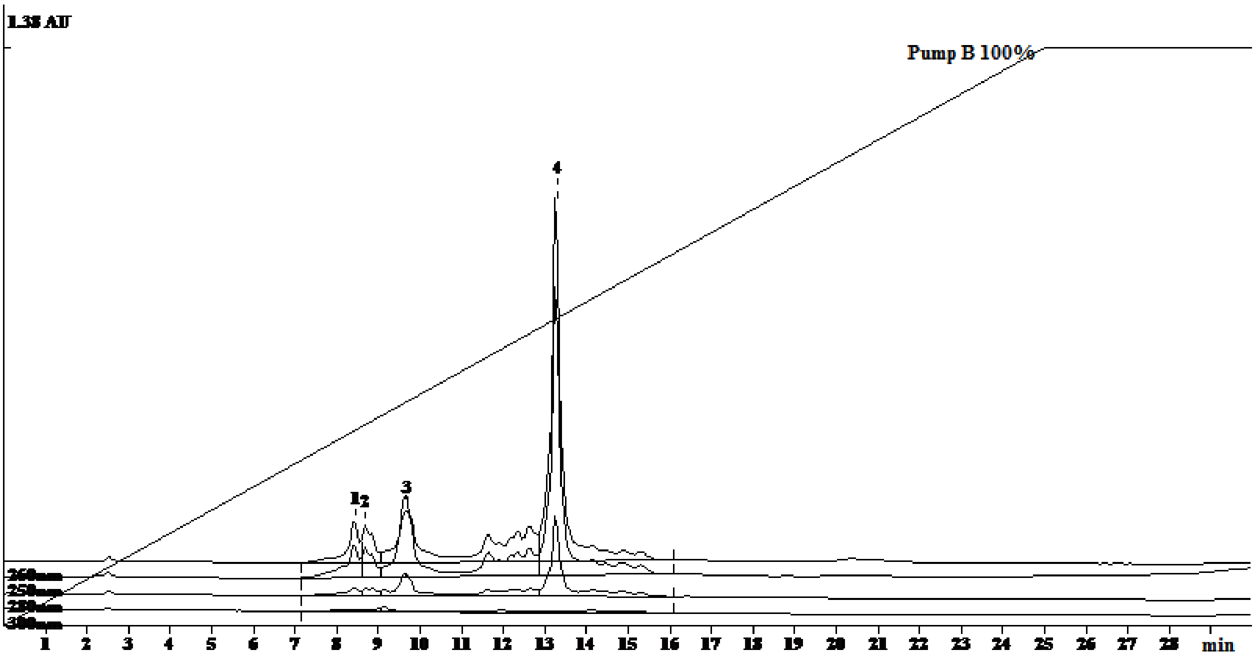

No	Retention	Height	Area
	µl	AU	AU*µl
1	844.77	0.10	3.025
2	871.46	0.09	2.741
3	968.58	0.12	15.775
4	1327.83	0.88	28.307
4	2999.21	1.20	49.848

No	Retention	250nm	280nm	300nm
	µl		(/260 nm)	
1	844.77	0.837	0.187	0.011
2	871.46	0.832	0.190	0.007
3	968.58	1.611	0.370	-0.013
4	1327.83	0.847	0.255	0.027

RPC HPLC trace of the reaction mixture after ammonia treatment of the sample of the support 6a after 4 cycles with monomer 1a (main product – 18a)

Report date:	03.04.2014 13:33:44
Analysis from:	19.10.2012 10:29:32
File:	10290010.CHW
Vial number:	1
Volume:	5.0 µl
Dilution:	1.00
COLUMN:	ProntoSIL-120-5-C18 AQ DB-2003
Size:	2.0 x 75 mm
Part.size:	5.0 µm
ELUENT:	0% CH_3CN 0.1M TEAAc pH7.0
	80%CH_3CN 0.1M TEAAc pH7.0
Flow:	100.00 µl/min
Temperature:	35.0°C
Pressure:	6.4 MPa

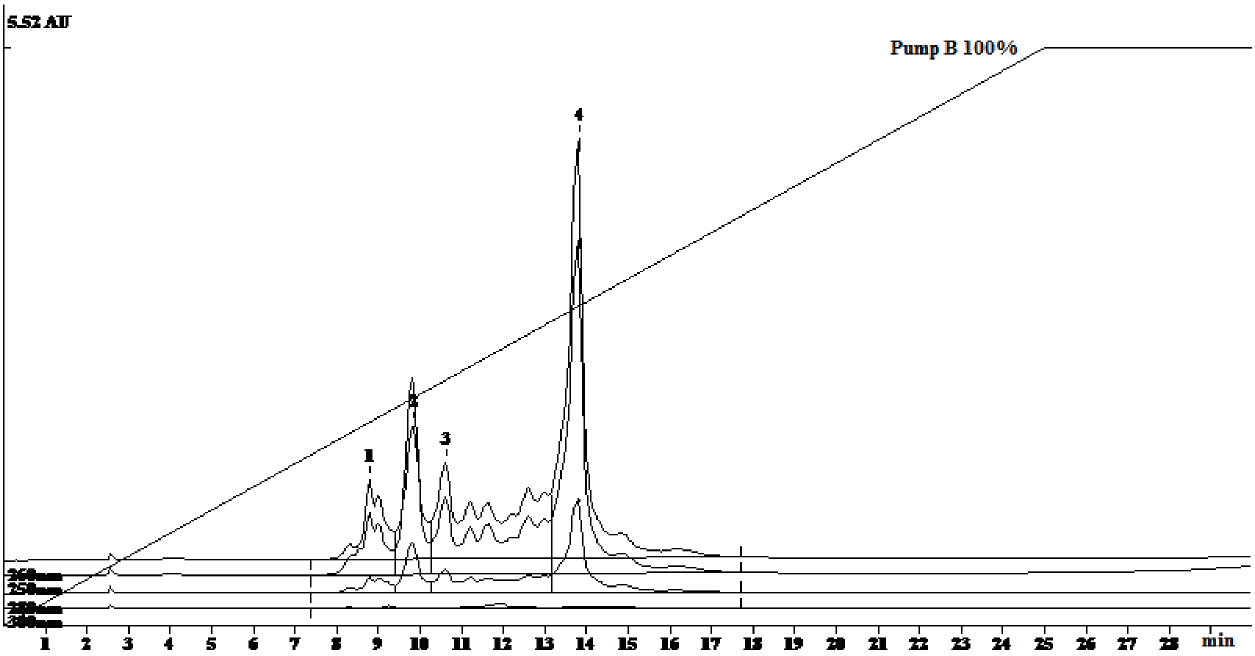

No	Retention	Height	Area
	µl	AU	AU*µl
1	881.43	0.76	42.297
2	984.98	1.29	55.315
3	1062.38	0.93	144.617
4	1381.68	4.02	216.732
4	2999.14	7.01	458.961

No	Retention	250nm	280nm	300nm
	µl		(/260 nm)	
1	881.43	0.926	0.238	0.045
2	984.98	1.462	0.365	-0.006
3	1062.38	0.810	0.218	-0.008
4	1381.68	0.815	0.240	0.022

RPC HPLC trace of the reaction mixture after ammonia treatment of the sample of the support 6d (main product – 2d)

Report date:	03.04.2014 13:53:04
Analysis from:	04.04.2012 13:35:58
File:	13350020.CHW
Vial number:	2
Volume:	5.0 µl
Dilution:	1.00
COLUMN:	ProntoSIL-120-5-C18 AQ DB-2003
Size:	2.0 x 75 mm
Part.size:	5.0 µm
ELUENT:	0% CH_3CN 0.1M TEAAc pH7.0
	80%CH_3CN 0.1M TEAAc pH7.0
Flow:	200.00 µl/min
Temperature:	35.0°C
Pressure:	5.5 MPa

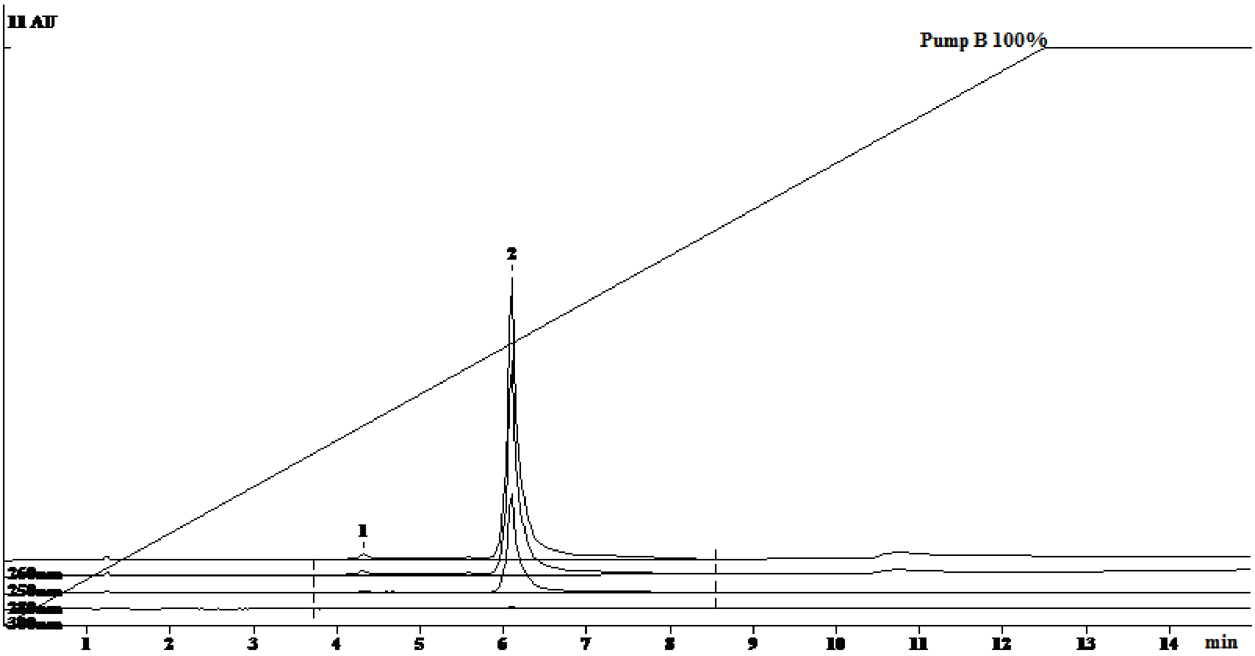

No	Retention µl	Height AU	Area AU*µl
1	864.98	0.13	14.981
2	1221.44	5.43	203.179
2	2999.39	5.56	218.160

No	Retention µl	250nm (/260 nm)	280nm	300nm
1	864.98	0.917	0.482	0.249
2	1221.44	0.769	0.363	0.012

RPC HPLC trace of the reaction mixture after ammonia treatment of the sample of the support 6d after 2 cycles with monomer 1d (main product – 16d)

Report date:	03.04.2014 13:51:58
Analysis from:	04.04.2012 13:16:15
File:	13160010.CHW
Vial number:	1
Volume:	5.0 µl
Dilution:	1.00
COLUMN:	ProntoSIL-120-5-C18 AQ DB-2003
Size:	2.0 x 75 mm
Part.size:	5.0 µm
ELUENT:	0% CH_3CN 0.1M TEAAc pH7.0
	80%CH_3CN 0.1M TEAAc pH7.0
Flow:	200.00 µl/min
Temperature:	35.0°C
Pressure:	6.7 MPa

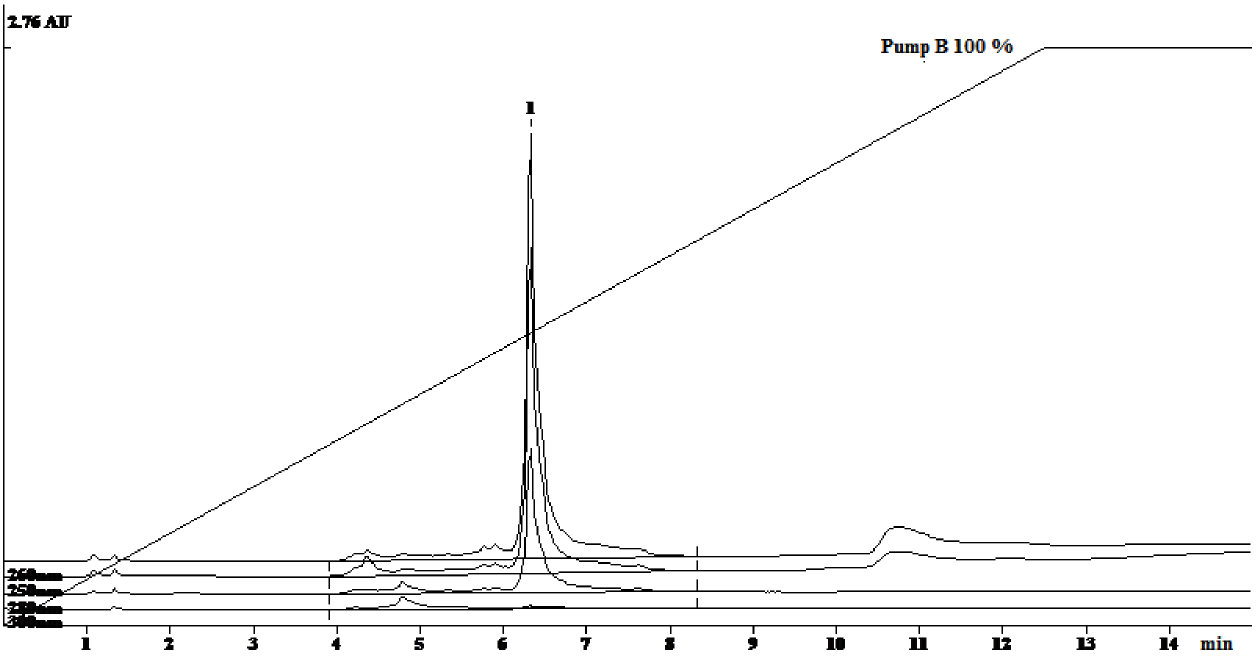

No	Retention	Height	Area
	µl	AU	AU*µl
1	1266.14	2.03	92.024

No	Retention	250nm	280nm	300nm
	µl	(/260 nm)		
1	1266.14	0.815	0.384	0.075

RPC HPLC trace of the reaction mixture after ammonia treatment of the sample of the support 6d after 4 cycles with monomer 1d (main product – 18d)

Report date:	03.04.2014 13:50:30
Analysis from:	12.09.2012 09:35:44
File:	09350010.CHW
Vial number:	1
Volume:	5.0 µl
Dilution:	1.00
COLUMN:	ProntoSIL-120-5-C18 AQ DB-2003
Size:	2.0 x 75 mm
Part.size:	5.0 µm
ELUENT:	0% CH_3CN 0.1M TEAAc pH7.0
	80%CH_3CN 0.1M TEAAc pH7.0
Flow:	100.00 µl/min
Temperature:	35.0°C
Pressure:	5.6 MPa

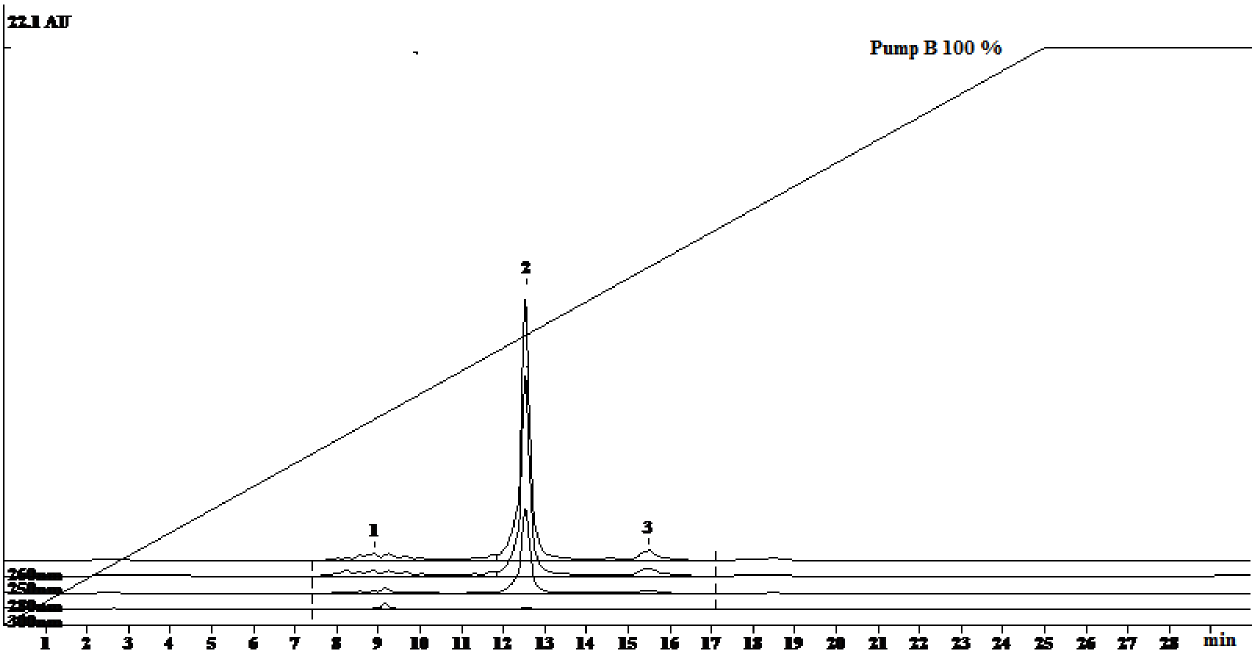

No	Retention	Height	Area
	µl	AU	AU*µl
1	890.97	0.29	42.170
2	1254.30	10.35	291.406
3	1551.36	0.38	26.103
3	2999.20	11.02	359.679

No	Retention	250nm	280nm	300nm
	µl	(/260 nm)		
1	890.97	0.895	0.400	0.124
2	1254.30	0.776	0.335	0.005
3	1551.36	0.820	0.361	0.015

RPC HPLC trace of the reaction mixture after ammonia treatment of the sample of the support 6e (main product – 2e)

Report date:	03.04.2014 13:41:01
Analysis from:	27.02.2013 13:38:24
File:	13380020.CHW
Vial number:	2
Volume:	5.0 µl
Dilution:	1.00
COLUMN:	ProntoSIL-120-5-C18 AQ DB-2003
Size:	2.0 x 75 mm
Part.size:	5.0 µm
ELUENT:	0% CH_3CN 0.1M TEAAc pH7.0
	80%CH_3CN 0.1M TEAAc pH7.0
Flow:	200.00 µl/min
Temperature:	35.0°C
Pressure:	3.3 MPa

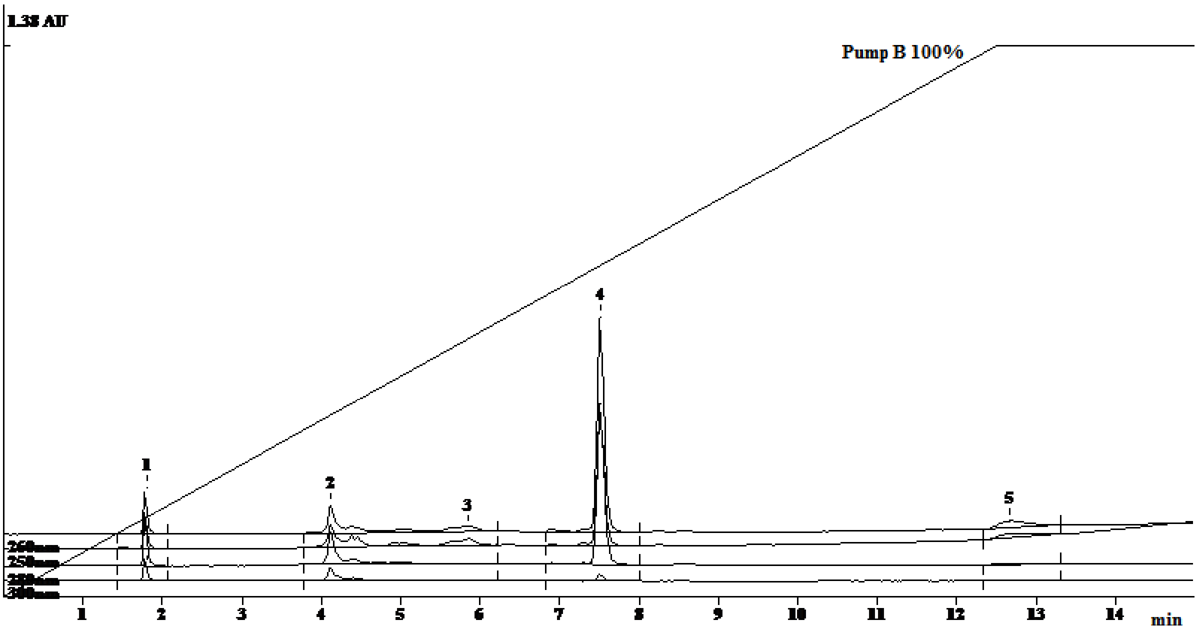

No	Retention µl	Height AU	Area AU*µl
1	358.65	0.13	1.203
2	825.54	0.07	2.928
3	1172.09	0.01	1.085
4	1503.23	0.54	11.753
5	2535.47	0.02	1.577

No	Retention µl	250nm	280nm (/260 nm)	300nm
1	358.65	0.904	1.127	0.457

2	825.54	1.130	0.976	0.380
3	1172.09	1.011	0.113	0.023
4	1503.23	0.651	0.740	0.032
5	2535.47	0.557	0.005	-0.004

RPC HPLC trace of the reaction mixture after ammonia treatment of the sample of the support 6e after 2 cycles with monomer 1e (main product – 16e)

Report date:	03.04.2014 13:47:59
Analysis from:	22.03.2013 15:51:00
File:	15510010.CHW
Vial number:	2
Volume:	5.0 µl
Dilution:	1.00
COLUMN:	ProntoSIL-120-5-C18 AQ DB-2003
Size:	2.0 x 75 mm
Part.size:	5.0 µm
ELUENT:	0% CH_3CN 0.1M TEAAc pH7.0
	80%CH_3CN 0.1M TEAAc pH7.0
Flow:	200.00 µl/min
Temperature:	35.0°C
Pressure:	4.7 MPa

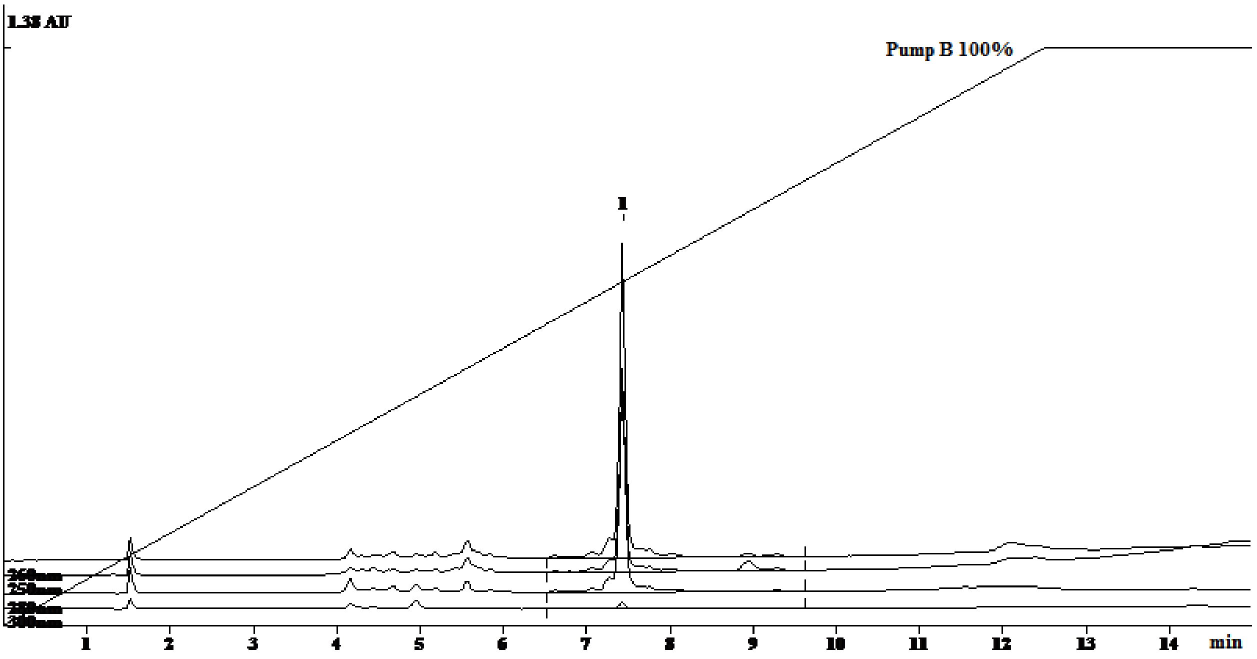

Quantitation method: Custom

No	Retention	Height	Area
	µl	AU	AU*µl
1	1488.41	0.79	16.706

No	Retention	250nm	280nm	300nm
	µl		(/260 nm)	
1	1488.41	0.679	0.683	0.028

RPC HPLC trace of the reaction mixture after ammonia treatment of the sample of the support 6e after 4 cycles with monomer 1e (main product – 18e)

Report date:	03.04.2014 13:39:47
Analysis from:	28.03.2013 13:51:11
File:	13510010.CHW
Vial number:	2
Volume:	5.0 µl
Dilution:	1.00
COLUMN:	ProntoSIL-120-5-C18 AQ DB-2003
Size:	2.0 x 75 mm
Part.size:	5.0 µm
ELUENT:	0% CH_3CN 0.1M TEAAc pH7.0
	80%CH_3CN 0.1M TEAAc pH7.0
Flow:	200.00 µl/min
Temperature:	35.0°C
Pressure:	4.5 MPa

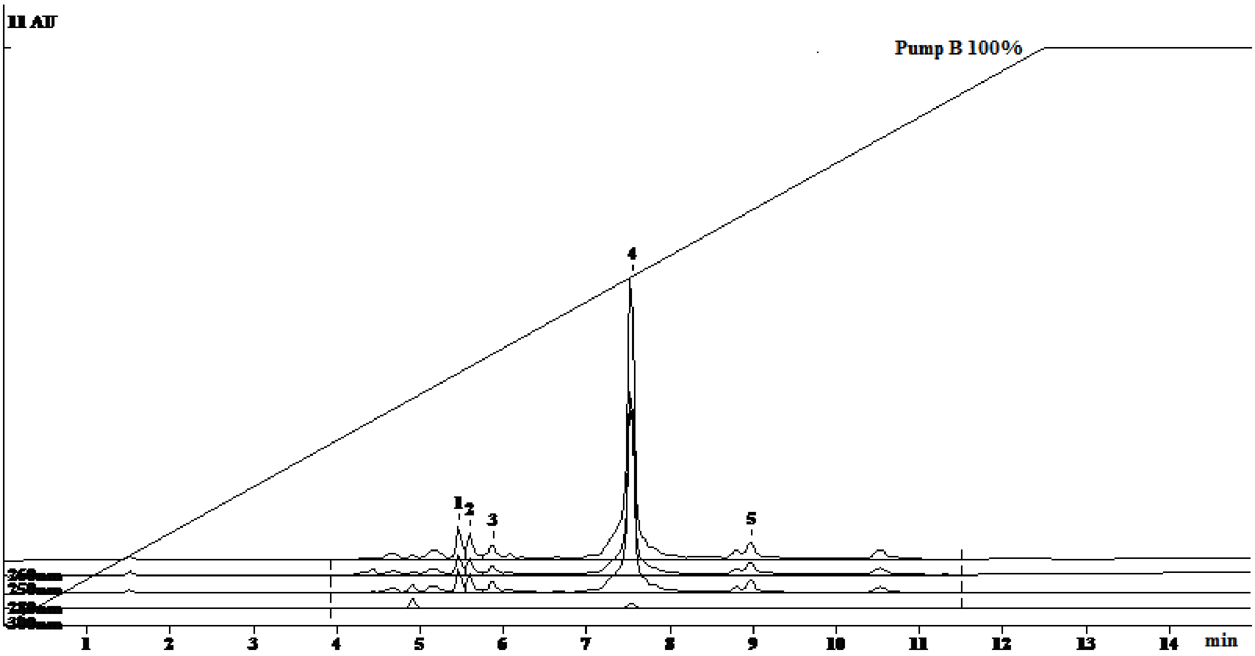

Quantitation method: Custom

No	Retention µl	Height AU	Area AU*µl
1	1094.85	0.67	29.282
2	1121.37	0.53	10.745
3	1176.37	0.32	16.046
4	1508.62	5.44	162.261
5	1796.46	0.34	30.412

No	Retention µl	250nm	280nm (/260 nm)	300nm
1	1094.85	0.666	0.676	0.017
2	1121.37	0.705	0.705	0.021

3	1176.37	0.659	0.698	0.043
4	1508.62	0.656	0.677	0.026
5	1796.46	0.679	0.639	0.023

RPC HPLC analysis of purified oligomer 7a

Report date:	03.04.2014 13:32:09
Analysis from:	31.10.2012 15:44:22
File:	15440010.CHW
Vial number:	1
Volume:	5.0 µl
Dilution:	1.00
COLUMN:	ProntoSIL-120-5-C18 AQ DB-2003
Size:	2.0 x 75 mm
Part.size:	5.0 µm
ELUENT:	0% CH_3CN 0.1M TEAAc pH7.0
	80%CH_3CN 0.1M TEAAc pH7.0
Flow:	100.00 µl/min
Temperature:	35.0°C
Pressure:	5.4 MPa

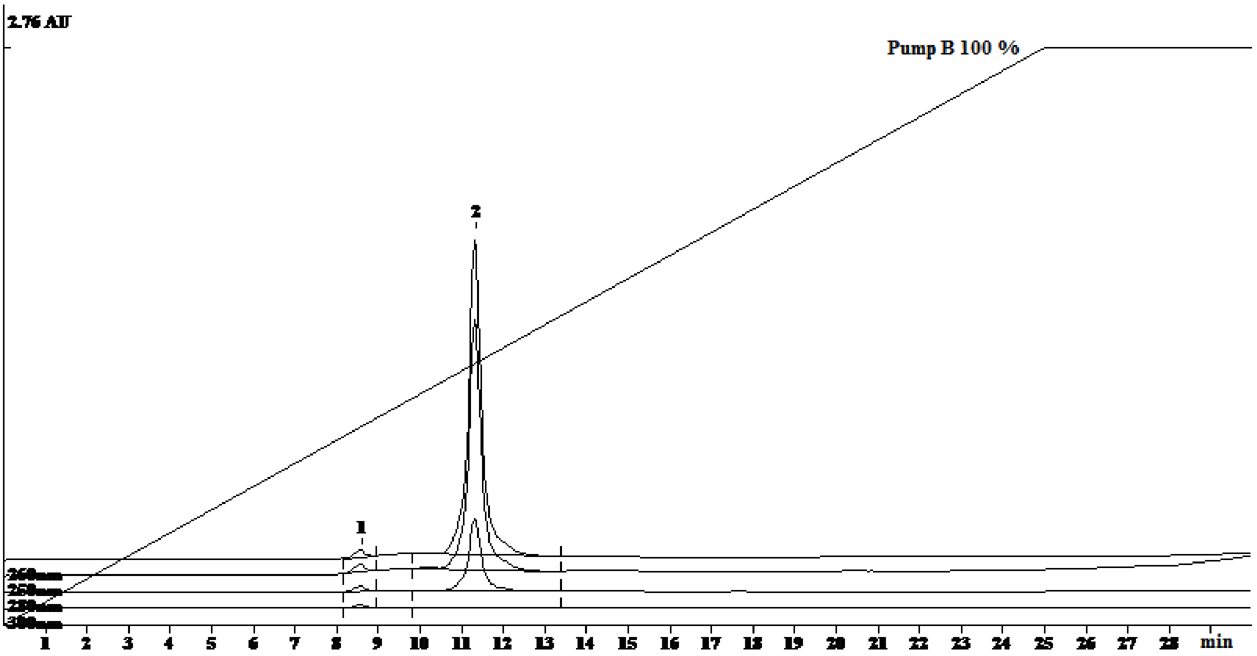

No	Retention µl	Height AU	Area AU*µl
1	859.14	0.04	0.967
2	1134.17	1.53	59.786
2	2999.13	1.57	60.753

No	Retention µl	250nm	280nm (/260 nm)	300nm
1	859.14	1.147	0.705	0.423
2	1134.17	0.791	0.230	-0.002

RPC HPLC analysis of purified oligomer 7d

Report date:	03.04.2014 13:49:32
Analysis from:	26.09.2012 12:44:25
File:	12440010.CHW
Vial number:	1
Volume:	5.0 µl
Dilution:	1.00
COLUMN:	ProntoSIL-120-5-C18 AQ DB-2003
Size:	2.0 x 75 mm
Part.size:	5.0 µm
ELUENT:	0% CH_3CN 0.1M TEAAc pH7.0
	80%CH_3CN 0.1M TEAAc pH7.0
Flow:	100.00 µl/min
Temperature:	35.0°C
Pressure:	5.5 MPa

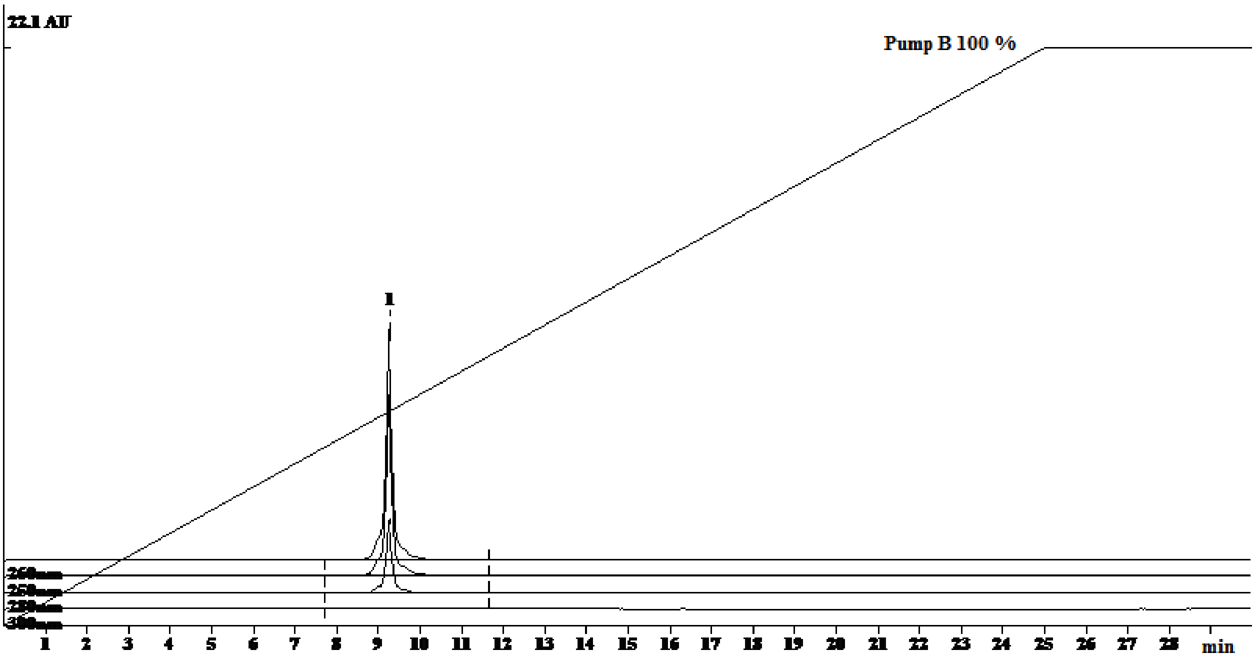

No	Retention µl	Height AU	Area AU*µl
1	927.66	9.16	180.083

No	Retention µl	250nm	280nm (/260 nm)	300nm
1	927.66	0.788	0.326	0.009

RPC HPLC analysis of purified oligomer 7e

Report date: 03.04.2014 13:17:27
Analysis from: 03.04.2013 15:14:33
File: 15140010.CHW
Vial number: 1
Volume: 5.0 µl
Dilution: 1.00
COLUMN: ProntoSIL-120-5-C18 AQ DB-2003
Size: 2.0 x 75 mm
Part.size: 5.0 µm
ELUENT: 0% CH_3CN 0.1M TEAAc pH7.0
80%CH_3CN 0.1M TEAAc pH7.0
Flow: 200.00 µl/min

Temperature: 35.0°C

Pressure: 4.7 MPa

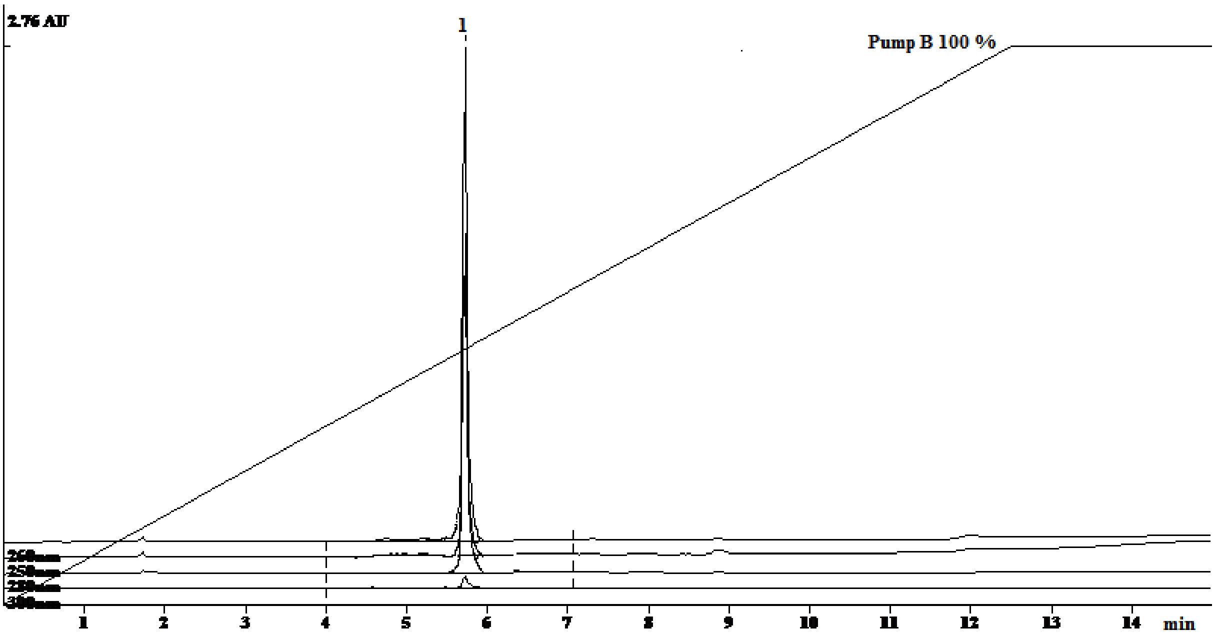

No	Retention µl	Height AU	Area AU*µl
1	1146.66	2.44	41.796

No	Retention µl	250nm	280nm (/260 nm)	300nm
1	1146.66	0.663	0.669	0.038
